# Radar-Based Detection of Obstructive Sleep Apnea: A Systematic Review and Network Meta-Analysis of Diagnostic Accuracy Across Frequency Bands

**DOI:** 10.3390/diagnostics15162111

**Published:** 2025-08-21

**Authors:** Nguyen Binh Minh Hoang Tran, Thi Quynh Trang Tran, Cheng-Yu Tsai, Jiunn-Horng Kang

**Affiliations:** 1 International Ph.D. Program in Medicine, College of Medicine, Taipei Medical University, Taipei 11031, Taiwan; d142113004@tmu.edu.tw (N.B.M.H.T.); d142111017@tmu.edu.tw (T.Q.T.T.); 2 Department of Rehabilitation, Ho Chi Minh City Hospital for Rehabilitation and Professional Diseases, Ho Chi Minh City 700000, Vietnam; 3 Faculty of Rehabilitation, University of Medicine and Pharmacy, Hue University, Hue 530000, Vietnam; 4 Division of Pulmonary Medicine, Department of Internal Medicine, Taipei Medical University-Shuang Ho Hospital, New Taipei City 23561, Taiwan; 5 TMU Research Center for Thoracic Medicine, Taipei Medical University, Taipei 11031, Taiwan; 6 Research Center of Sleep Medicine, College of Medicine, Taipei Medical University, Taipei 11031, Taiwan; 7 School of Respiratory Therapy, College of Medicine, Taipei Medical University, Taipei 11031, Taiwan; 8 Sleep Center, Taipei Medical University-Shuang Ho Hospital, New Taipei City 23561, Taiwan; 9 TMU Research Center of Artificial Intelligence in Medicine, Taipei Medical University, Taipei 11031, Taiwan; 10 School of Biomedical Engineering, College of Biomedical Engineering, Taipei Medical University, Taipei 11031, Taiwan; 11 Department of Physical Medicine and Rehabilitation, School of Medicine, College of Medicine, Taipei Medical University, Taipei 11031, Taiwan; 12 Department of Physical Medicine and Rehabilitation, Taipei Medical University Hospital, Taipei 11031, Taiwan; 13 Graduate Institute of Nanomedicine and Medical Engineering, College of Biomedical Engineering, Taipei Medical University, Taipei 11031, Taiwan

**Keywords:** obstructive sleep apnea, radar, network meta-analysis, frequency band

## Abstract

**Background**: Obstructive sleep apnea (OSA) is one of the most prevalent yet underdiagnosed sleep disorders. We evaluated the diagnostic accuracy of radar-based systems and ranked frequency bands for the non-contact detection of OSA. **Methods**: A systematic search of six databases was conducted from inception to May 23, 2025. Eligible studies included adults assessed for OSA using radar-based systems compared to polysomnography. Hierarchical SROC modeling, threshold-based meta-analyses, and frequency band-stratified network meta-analysis were performed. Certainty of evidence was assessed using GRADE. The PROSPERO registration number is CRD420251059236. **Results**: We identified 23,906 records and included 20 studies involving 1540 participants. The primary outcome included a high area under the curve (AUC) of approximately 0.91, an optimal apnea–hypopnea index (AHI) cutoff of ≥22 with a sensitivity of 0.8155 (95% confidence interval (CI): 0.6862–0.8993) and specificity of 0.8819 (95% CI: 0.7799–0.9402). At an AHI threshold of 30, X-band dual radar performed the best, followed by K-band, which yielded significant but more variable results. C-bands consistently showed lower diagnostic values. **Conclusions**: This study provides a novel radar band comparison for OSA detection, highlighting clinically relevant thresholds. Key limitations are indirect comparisons and limited, varied samples. Radar-based systems show high sensitivity for OSA detection, optimized by frequency, radar type, artificial intelligence support, and dual sensors within 0.2–1.5 m. Future work should expand the frequency analysis, standardize AHI thresholds, and validate results in specific subgroups.

## 1. Introduction

Sleep quality is connected to all-cause mortality [1,2] and various chronic conditions [3,4,5,6,7]. Among the most prevalent and underdiagnosed sleep-related disorders is obstructive sleep apnea (OSA) [5,8,9,10], which affects nearly 1 billion people worldwide [11]; alarmingly, up to 93% of middle-aged women and 82% of men with moderate to severe OSA remain undiagnosed [12]. OSA is linked to endothelial dysfunction and higher cardiovascular events in untreated patients [13,14,15] and even sudden cardiac death [16].

Given the underdiagnosis of OSA and limitations of polysomnography (PSG), including inaccessibility [17], conditional necessity [18], the need for repeat justification [19], or even the potential risk of infection from cross-contamination [20], non-contact sensing technologies offer a promising alternative [21]. In response, digital alerting systems have shown clinical benefits, including a 9.6% decrease in hospitalizations, an average reduction of 1.043 days of hospital stays, and a 3% decrease in all-cause mortality [22]. Among these unobtrusive monitoring technologies, radar, operating at 120 GHz (millimeter-wave), has shown superior performance over ballistocardiography, with heart rate errors of just 0.4 bpm [20]. It also outperformed phonocardiography in heartbeat timing accuracy, with a lower root mean square error of 44.2 ms [23]. Terahertz-wave radar plethysmography provides stronger and cleaner pulsatile signals than remote photoplethysmography [24]. Unlike infrared and thermography, which are susceptible to surface heat, environmental light, skin emissivity, and clothing, radar can reliably function through garments under variable light or air conditions and also maintain stability during movement [25,26]. Radar types, including continuous wave (CW), frequency-modulated CW (FMCW), impulse radio ultra-wideband (UWB), and step frequency CW (SFCW), vary in cost, frequency range, depth penetration, spatial resolution, and noise tolerance [21,27,28,29]. Proper frequency allocation is essential, in accordance with Federal Communications Commission (FCC) regulations and common industrial, scientific, and medical (ISM) bands [28,29]. To date, vital-sign radar types span broad Institute of Electrical and Electronics Engineers (IEEE) bands [30], from very high frequency (VHF) to sub-terahertz [31,32,33,34,35,36,37,38].

Despite a growing body of research, it remains unclear which radar frequency bands have the highest diagnostic validity when benchmarked against PSG in clinical settings. A concerning limitation observed in several pilot studies validating radar-based tools for OSA has been the omission of critical technical specifications, particularly the operating frequency of the radar device employed [39,40,41], instead of describing the device by its brand or commercial model, resulting in context-deprived diagnostic metrics. A previous review by Khalil et al. [42] broadly examined various non-contact technologies, such as audio and computer vision, whereas Boiko et al. generally discussed sensor types [21,42]. Our review addresses this gap by evaluating how different radar frequency bands influence detection of the apnea–hypopnea index (AHI) and the overall radar-based diagnostic performance. Understanding this frequency-dependent performance is crucial, as it significantly impacts key clinical outcomes, including the sensitivity, specificity, and accuracy of AHI classification [43,44]. Therefore, in this work, we aimed to assess the diagnostic accuracy of radar-based sleep monitoring systems across various frequency bands, using standardized AHI thresholds, to identify which frequency bands provide the most-reliable evaluation of sleep-disordered breathing.

## 2. Materials and Methods

We conducted a systematic review and network meta-analysis of diagnostic test accuracy studies comparing non-contact radar-based systems with the reference gold standard of PSG for detecting OSA. This review followed PRISMA (Preferred Reporting Items for Systematic Reviews and Meta-Analyses) guidelines [45,46], and the *Cochrane Handbook for Systematic Reviews of Diagnostic Test Accuracy* [47,48] (Tables S1 and S2). It was also registered with PROSPERO (registration no. CRD420251059236).

### 2.1. Search Strategy and Selection Criteria

**Figure 1:**
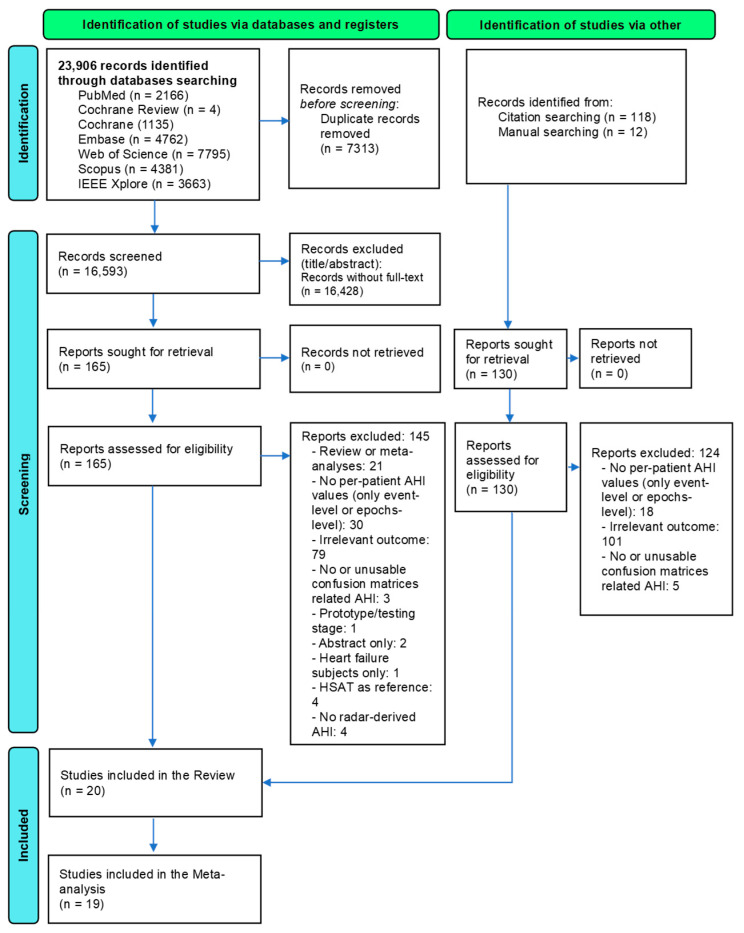
PRISMA flowchart of all included studies.

A systematic search was conducted across six electronic databases: PubMed (U.S. National Library of Medicine), Embase (Excerpta Medica Database, Elsevier), Scopus (Elsevier), the Cochrane Library (Wiley), Web of Science Core Collection (Clarivate Analytics), and IEEE Xplore Digital Library (Institute of Electrical and Electronics Engineers), from inception to 23 May 2025, without language restrictions. Reference lists of included articles and related systematic reviews were also screened to identify additional relevant studies. The flow chart illustrating the selection process for this study is presented in Figure 1. In addition to peer-reviewed literature, our search strategy did not exclude conference abstracts, proceedings, or preprints across all six databases, with IEEE Xplore–well recognized for its extensive coverage of technology-related grey literature, ensuring the inclusion of such sources to further mitigate potential publication bias. The details and search strategy, including the search terms, are provided in Tables S3 and S4 and Supplementary Materials. Two reviewers independently screened titles, abstracts, and full texts to determine eligibility, resolving any discrepancies through discussions with a third reviewer. If multiple publications reported results from the same population or device, only the version with the most comprehensive and recent data was included.

### 2.2. Inclusion and Exclusion Criteria

Diagnostic performance studies were included if they enrolled adults suspected of having OSA and evaluated non-contact radar-based monitoring systems designed to detect or classify OSA, using a reference standard such as PSG. Eligible studies had to report sufficient data to extract or calculate quantifiable outcomes such as sensitivity, specificity, area under the curve (AUC), accuracy, positive predictive value (PPV), negative predictive value (NPV), or confusion matrix elements (true positives (TPs), false positives (FPs), true negatives (TNs), and false negatives (FNs)) at different AHI thresholds. Reviews, abstracts, and prototype-only reports were excluded, as were studies limited to non-representative subgroups or studies lacking per-patient AHI data or AHI calculated over the full recording, radar-derived AHI measurements, and outcomes relevant to an OSA diagnosis, to ensure consistency and generalizability.

### 2.3. Data Extraction and Analysis

Data extraction was conducted independently by two reviewers using a standardized form, with disagreements addressed by consensus or consultation with a third reviewer. Extracted data included study characteristics (such as author, year, and country), radar system specifications (including frequency band, technique, and device type), participant details (such as sample size and population type), and diagnostic outcomes (TPs, FPs, TNs, and FNs) at various AHI thresholds (≥5, ≥10, ≥15, ≥20, ≥ 25, and ≥30 events/h), pooled only when multiple studies used the same cutoff. When raw counts of apneas, hypopneas, and total sleep time were available, the AHI was calculated using the AASM-recommended formula for consistent thresholds [49].

Analyses were performed with R statistical software (v2025.05.0+496; R Foundation, Vienna, Austria). A hierarchical summary receiver operating characteristic (HSROC) model (using the diagmeta package) with a common intercept and slope was applied to log-transformed AHI cutoffs to generate summary ROC (SROC) curves and identify optimal thresholds. Additionally, a bivariate meta-analysis (using the mada package) was performed to estimate pooled sensitivity and specificity across AHI thresholds (of ≥5, ≥15, and ≥30 events/h), incorporating continuity correction and Wilson confidence intervals. Small-study effects and publication bias were approximately evaluated using funnel plots by Deeks [50]. A pairwise random-effects meta-analysis was conducted (using the metagen package) for AHI ≥ 30 events/h and stratified by frequency bands. A network meta-analysis (using the netmeta package) compared the log diagnostic odds ratios of various radar bands and types against PSG at an AHI threshold of ≥30 events/h based on studies reporting specific radar modalities. Inconsistency was evaluated using the design-by-treatment interaction model, partitioning Q into within- and between-design components, with τ^2^, τ, and I^2^ used to quantify heterogeneity. Comprehensive information about the data analysis is available in Tables S5–S25, Figures S1–S10 and Supplementary Materials. Risk of bias assessments were conducted using the QUADAS-2 tool, and the GRADE method for evaluating the certainty of evidence adhered to recommendations outlined in the Cochrane Handbook [51,52]. Sensitivity tests were conducted by replacing data based on standardized AHI thresholds with those derived from data-driven or model-optimized cutoffs or by substituting results from the best-performing models with those from alternative models provided in the same studies. In addition, the analysis was repeated after excluding data from one preprint study.

## 3. Results

### 3.1. Study Selections and Quality Assessment

We identified 23,906 records in total through database searches and assessed 295 full-text articles for eligibility. Ultimately, 20 studies from the published literature were included, which involved 1540 participants and were published between 2012 and 2025. These studies evaluated the diagnostic accuracy of various non-contact radar systems compared to PSG across multiple frequency bands. A summary of the included studies and their characteristics is provided in Table 1 and Tables S26–S28.

**Figure 2:**
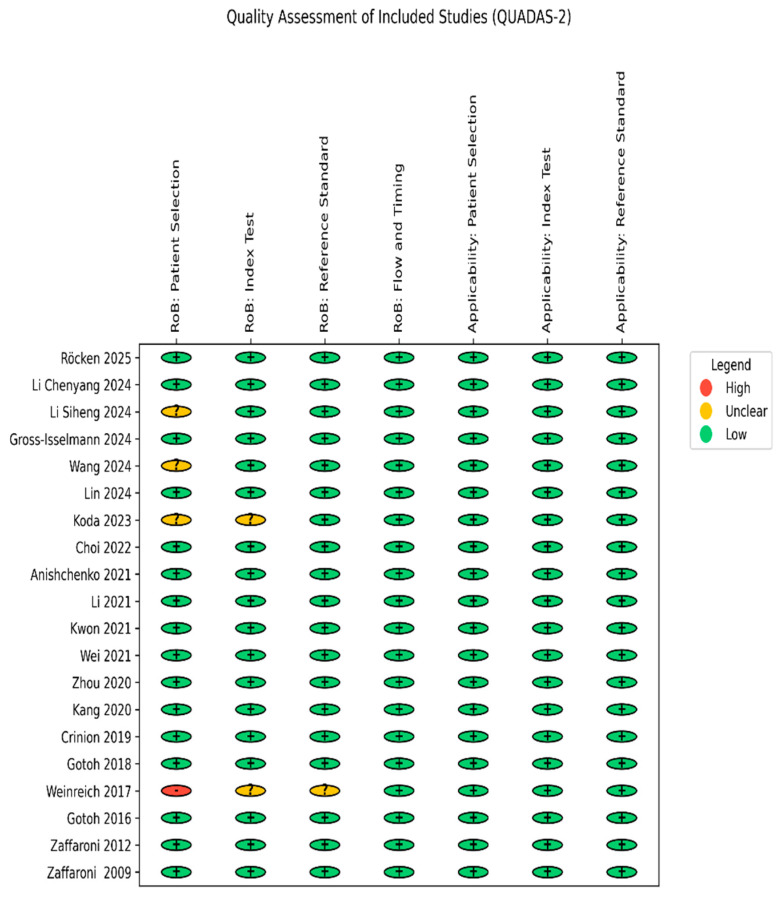
Quality assessment of the studies (QUADAS-2 tool) [39,40,41,43,53,54,55,57,58,59,60,61,62,63,64,65,66,67,68,69].

Risk of bias assessments are detailed in Tables S27 and S28. Most studies demonstrated a low risk of bias, although unclear patient selection and reporting were noted in a few (Figure 2). One particular study expressed concerns about selection and interpretation methods [53]. Eight studies lacked funding disclosure; six had industry affiliations, and two were industry funded. Language bias was possible in one Japanese and one Chinese study. Results using GRADE to assess the certainty of evidence are shown in Table 2 and also detailed in Tables S27 and S28. One small-sample, pilot study was excluded from the network meta-analysis [54]. One preprint study was evaluated for inclusion due to its methodological completeness, detailed reporting, and comprehensive presentation of AHI-level results [55,56].

**Table 1:** Summary of included studies on radar-based sleep apnea detection: study characteristics, radar configurations, and validation methods.

Lead Author and Year	Design	Country	Mean Age (Years)	Sex (% Male)	Radar				Comparison	Target	Total N	Purpose	Classification System or Algorithm	Distance (Sensor–Bed/Participant)
					Type	Band	Frequency	Setup						
Zaffaroni 2009 [57]	Cross-sectional	Ireland	53.9	82.2	Pulsed	C	5.8 GHz	Single	PSG	OSA	157	Algorithm development and initial validation	Proprietary software, including sleep/wake algorithm	0.2 m from subject, 0.5 m elevation from bed edge
Zaffaroni 2012 [58]	Cross-sectional	Ireland	49.9	79.7	Pulsed	C	5.8 GHz	Single	PSG	OSA	75	Clinical performance validation	Proprietary software, including sleep/wake algorithm, and respiratory envelope analysis	≤1.5 m
Gotoh 2016 [59]	Cross-sectional	Japan	49.8	80	CW	X	10.525 GHz	Dual	PSG, SpO_2_	OSA	20	Clinical performance validation	Event detection based on respiratory amplitude change and phase angle filtering; moving average baseline comparison	Two radar sensors placed under mattress, approx. 30 cm below shoulders, and 20 cm laterally from center line
Wein-reich 2017 [53]	Cross-sectional	Germany	56.4	80.7	Pulsed	C	5.8 GHz	Single	PSG	OSA, PLMS, CSR	57	Clinical validation in detecting combined SDB and PLMS using SDI as a unified index	Movement-based analysis using Doppler phase shifts; SDI (AHI and PLMI); rule-out screening approach	Device placed <1 m from bed, 0.25–0.5 m above mattress, aimed at the torso
Gotoh 2018 [60]	Cross-sectional	Japan	49	81.5	CW	X	10.525 GHz	Dual	PSG	OSA	27	Clinical validation with adaptive hypopnea threshold optimization	Custom rule-based algorithm with ROC-based K-value optimization for hypopnea threshold	Sensors placed beneath mattress, each 20 cm left and right from the body midline, near the iliac bone
Crinion 2019 [41]	Cross-sectional	Ireland	54.7	84.4	Pulsed	C	5.8 GHz	Single	PSG, HSAT	OSA, Hypertension	125	Clinical validation in both sleep clinic and hypertensive populations	Proprietary signal processing software (non-AI), includes respiration and motion analysis to estimate the AHI	Device placed on bedside table, approximately 1 m from patient
Kang 2020 [43]	Cross-sectional	South Korea	45.7	80.9	IR-UWB	C	6.5–8.0 GHz	Single	PSG	OSA	99	Development and validation of an IR-UWB radar algorithm	constant false alarm rate (CFAR) algorithm with additional weight function adaptation; developed using MATLAB	0.5 m from the head
Zhou 2020 [61]	Cross-sectional	China	38.1	71	UWB	C	6–8 GHz	Single	PSG	OSA	176	Clinical performance validation	Embedded chip in radar for automatic AHI calculation (based on respiratory motion and body movement signals)	1.5 m from patient, placed 15–25 cm above mattress on a bedside table
Anish-chenko 2021 [62]	Cross-sectional	Russia	51.3	64.5	NR	K	24.0 and 24.1 GHz	Dual	PSG	OSA	31	Clinical performance validation	Ensemble ML classifier (Gentle Boost) trained on time/frequency features and entropy/Lyapunov measures from radar signals	Two radars: BRL1: 1.5 m lateral from bed, 1.2 m above floor, BRL2: wall-mounted above bed, 1.6 m high; both targeting chest
Kwon 2021 [63]	Cross-sectional	South Korea	38.7	61.1	IR-UWB	C	fc = 7.29 GHz, BW = 1.5 GHz	Single	PSG	OSA	36	Clinical validation of real-time AHI estimation using radar and deep learning without handcrafted features	Hybrid deep learning: CNN and BiLSTM for segment classification, with sliding 20 s window, and event detector based on consecutive AH-labeled segments	0.84–1.32 m (mean 0.98 ± 0.31 m), placed on tripod facing chest
Li 2021 [39]	Cross-sectional	China	47.8	80.3	NR	NR	NR	Single	PSG	OSA	71	Clinical performance validation	Built-in automatic analysis system in radar device	1 m away from the body; radar placed beside bed, with fingertip oxygen ring attached
Wei 2021 [40]	Cross-sectional	China	43	83.6	UWB	C	6–8 GHz	Single	PSG	OSA	67	Clinical performance validation of a novel UWB radar device combined with a pulse oximeter ring	Fully automated analysis by the UWB device; uses respiratory motion and blood oxygen signals	1 m from edge of bed, 0.5 m height, aligned with subject’s chest
Choi 2022 [64]	Cross-sectional	South Korea	53.5	56.8	FMCW	V	60 GHz	Single	PSG	OSA	44	Clinical performance validation combined with deep learning	Deep learning: convolutional recurrent neural network (CRNN)	2 m, ceiling-mounted above patient’s chest
Koda 2023 [54]	Cross-sectional	Japan	NR	NR	FMCW	W	79 GHz	Single	PSG	OSA	5	Development of a radar-based, non-contact system using EM algorithm	Expectation–maximization (EM) algorithm on radar-derived respiratory displacement amplitude (non-AI, unsupervised statistical model)	1.5 m (from radar echoes and hospital room setup); radar mounted to capture full body motion via array imaging
Lin 2024 [65]	Cross-sectional	Taiwan	44.8	74.5	CW	K	24 GHz	Single	PSG	OSA	196	Development and validation of a non-contact 24-GHz radar system with deep learning (DL)	Hybrid DL (deep neural decision trees); machine learning techniques for respiratory event and sleep stage classification	1–1.5 m
Gross-Isselmann 2024 [66]	Cross-sectional	Germany	51.98	57	CW	K	24 GHz	Single	PSG	OSA	141	Performance validation in clinical and home environs	Proprietary automatic scoring algorithm (not DL) with optional SpO_2_ integration	50 cm from thorax, mounted beside the bed slightly above mattress level
Wang 2024 [55]	Cross-sectional	China	NR	NR	FMCW	V	fc = 60 GHz, BW = 3 GHz	Single	PSG	OSA	100	Development and validation of ROSA—a radar plus SpO_2_ fusion system combined with deep learning	Deep learning: RASA R-CNN for event detection, RassNet for sleep staging, soft fusion of radar and SpO_2_	Radar mounted above the head of the bed, facing the chest
Li-Chenyang 2024 [67]	Cross-sectional	China	35.3	51.7	NR	mm	NR	Single	PSG	OSA	155	Clinical performance validation	Signal fusion of radar and oximeter data using ML-based classification algorithms	Device placed beside bed in sleep lab
Li-Siheng 2024 [68]	Cross-sectional	China	NR	76	IR-UWB	C	fc = 7.3 GHz, BW = 1.4 GHz	Single	PSG	OSA	18	Development and validation of Respnea, a non-intrusive, fine-grained respiration monitoring system using radar and DL	CNN-based encoder, multi-head self-attention, contrastive learning; AI-based model	40–100 cm (optimal range tested); device placed on nightstand beside bed
Röcken 2025 [69]	Cross-sectional	Switzer-land	55.3	60.8	CW	K	24 GHz	Single	PSG	OSA	102	Clinical performance validation	Proprietary signal processing by manufacturer	40–50 cm

AH, apnea–hypopnea; AHI, apnea–hypopnea index; AI, artificial intelligence; BiLSTM, bidirectional long short-term memory; BRL, bioradiolocation; BW, bandwidth; CNN, convolutional neural network; CSR, Cheyne–Stokes respiration; CW, continuous wave; fc, center frequency; FMCW, frequency-modulated continuous wave; HSAT, home sleep apnea test; IR-UWB, infrared ultra-wide band; ML, machine learning; NR, not reported in the original study and could not be inferred from the available device or methodological descriptions; PLMI, period limb movement index; PLMS, periodic limb movements in sleep; PSG, polysomnography; RASA R-CNN, radar-based sleep apnea (detection) using region-based convolutional neural network; ROC, receiver operating characteristic; SDB, sleep disordered breathing; SDI, sleep disorder index.

**Table 2:** Summary statistics and certainty of evidence for radar-based OSA detection across AHI thresholds.

AHI Threshold	Test Accuracy Summary	Participants (*n* Studies)	Subgroup Results	Results per 1000 Patients (95% CI)	Factors that May Decrease Certainty of the Evidence	Certainty of Evidence (GRADE)
AHI ≥ 5	Pooled sensitivity: 0.944 (0.912–0.964) Pooled specificity: 0.699 (0. 519–0. 833) AUC: 0.941 I^2^ = 10.7%	1435 (18)	Prevalence: 77.42%		Risk of bias: Not serious; Indirectness: Not serious; Inconsistency: Not serious; Imprecision: Not serious Publication bias: Strongly detected	⊕⊕⊕⊝ Moderate
			TPs: 1055 FNs: 56 TNs: 233 FPs: 91	TPs: 731 (706–747) FNs: 44 (28–68) TNs: 158 (117–188) FPs: 68 (38–109)		
AHI ≥ 15	Pooled sensitivity: 0.879 (0.827–0.916) Pooled specificity: 0.897 (0.818–0. 944) AUC: 0.935 I^2^ = 12.8%	1468 (18)	Prevalence: 51.57%		Risk of bias: Not serious; Indirectness: Not serious; Inconsistency: Not serious; Imprecision: Not serious Publication bias: Strongly detected	⊕⊕⊕⊝ Moderate
			TPs: 678 FNs: 79 TNs: 631 FPs: 80	TPs: 453 (426–473) FNs: 63 (43–89) TNs: 434 (396–457) FPs: 50 (27–88)		
AHI ≥ 30	Pooled sensitivity: 0.827 (0.699–0.908) Pooled specificity: 0.950 (0.900–0.976) AUC: 0.957 I^2^ = 17.8%	1289 (15)	Prevalence: 32.12%		Risk of bias: Not serious; Indirectness: Not serious; Inconsistency: Not serious; Imprecision: Not serious Publication bias: Strongly detected	⊕⊕⊕⊝ Moderate
			TPs: 347 FNs: 67 TNs: 832 FPs: 43	TPs: 266 (224–292) FNs: 56 (30–97) TNs: 645 (611–662) FPs: 34 (16–68)		

I^2^ estimates: Zhou and Dendukuri approach. OSA, obstructive sleep apnea; AHI, apnea–hypopnea index; AUC, area under the curve; TPs, true positives; FNs, false negatives; TNs, true negatives; FPs, false positives.

### 3.2. Study Characteristics

All included studies primarily targeted OSA, with some also addressing periodic limb movements in sleep (PLMS) or sleep staging, with mean participant ages ranging from 35 to 56 years, males representing 51.7–84.4%, and sample sizes ranging from 5 to 196. Most studies utilized the C band (nine studies), followed by the K band (four studies), with fewer studies using the X, V, W band, and mmWave. Radar types varied, with pulsed (four studies), continuous wave (CW) (five), frequency-modulated CW (FMCW) (three), ultra-wide bandwidth (UWB) (one), and infrared (IR)-UWB (four) represented, mostly in single-sensor setups. Sensor distances varied from 20 cm to 2 m, with placements on the bedside under the mattress, on a tripod, or mounted on the wall/ceiling, typically targeting the chest. These studies employed diverse classification approaches, ranging from proprietary and rule-based algorithms to advanced machine learning (ML) and deep learning (DL) models.

### 3.3. Summary Statistics

Using the GRADE framework, the certainty of evidence supporting radar-based detection of OSA was rated as moderate to high (Table 2). Across all evaluated AHI thresholds (≥5, ≥15, and ≥30 events/h), no serious concerns were identified regarding the risk of bias, indirectness, inconsistency, or imprecision. However, publication bias was strongly suspected and also supported by Deeks’ funnel plot and test *p* values (Tables S6–S11, Figures S2–S4), which led to a downgrade in the certainty of the evidence.

**Figure 3:**
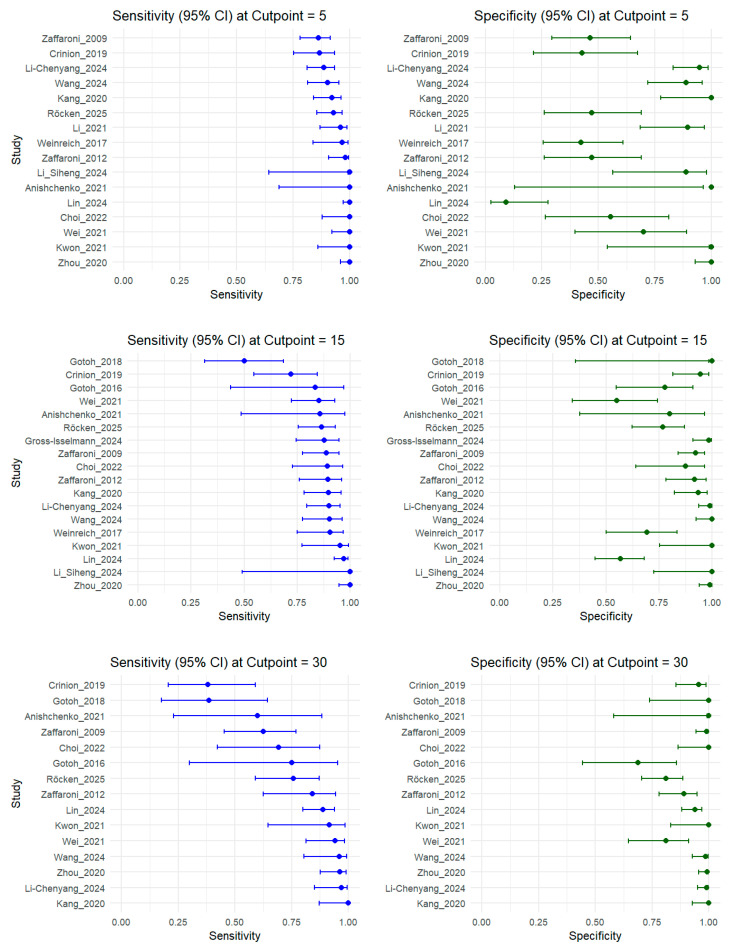
Coupled forest plot of the sensitivity and specificity at three apnea–hypopnea index (AHI) thresholds [39,40,41,43,53,54,55,57,58,59,60,61,62,63,64,65,66,67,68,69].

**Figure 4:**
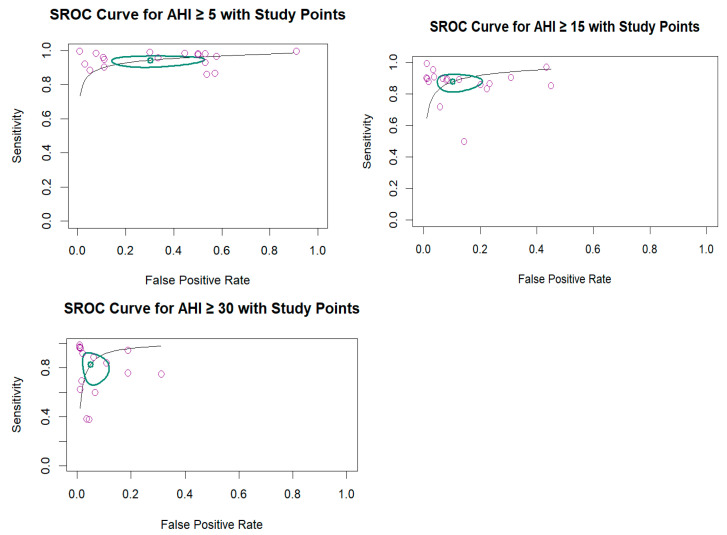
Summary receiver operating characteristic (SROC) curves based on AHI thresholds. The purple circles represent individual studies included in the analysis.

The pooled diagnostic accuracy of radar-based devices across three AHI thresholds demonstrated consistently high performances (Figure 3 and Figure 4). With data of 1435 participants from 18 studies at an AHI of ≥5 events/h, sensitivity was highest at 0.944 (95% confidence interval (CI): 0.912–0.964), and specificity was 0.699 (95% CI: 0.519–0.833), with an AUC of 0.941 and low heterogeneity (I^2^ = 10.7%). Analysis of AHI ≥15 events/h, including 1468 patients from 18 studies, showed a sensitivity of 0.879 (95% CI: 0.827–0.916) and a specificity of 0.897 (95% CI: 0.818–0.944), yielding an AUC of 0.935 and moderate heterogeneity (I^2^ = 12.8%). In 15 studies with data involving 1289 cases at an AHI of ≥30 events/h, specificity increased to 0.950 (95% CI: 0.900–0.976), while sensitivity decreased to 0.827 (95% CI: 0.699–0.908), resulting in the highest AUC of 0.957 (I^2^ = 17.8%).

### 3.4. Multiple Cutoffs Model

**Figure 5:**
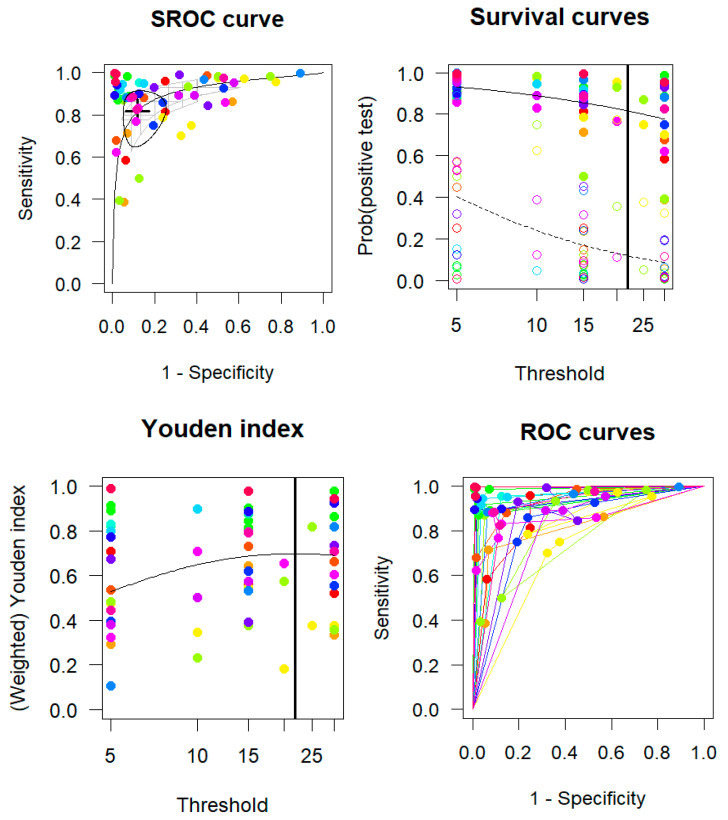
Diagnostic accuracy summary plots with multiple cutoffs. Summary of the receiver operating characteristic (SROC) curve. Survival plot illustrating the rates of positive test results for individuals with and without obstructive sleep apnea (OSA), represented by solid and hollow circles, across various thresholds. The Youden index is displayed with the threshold values. Study-specific ROC curves. Points or lines of the same color correspond to the same study.

The multiple thresholds meta-analysis of 19 studies (61 cutoffs across six AHI thresholds) identified an optimal AHI cutoff of 21.91 events/h, with sensitivity of 0.8155 (95% CI: 0.6862–0.8993) and specificity of 0.8819 (95% CI: 0.7799–0.9402) (Figure 5). A clear inverse relationship was observed between the sensitivity and specificity as the AHI threshold increased. Importantly, the CIs for both metrics remained relatively wide across thresholds. The common intercept, common slope model showed a high AUC, with 0.9105 (95% CI: 0.8353–0.9551) based on the confidence region for sensitivity given the specificity, and 0.9104 (95% CI: 0.8576–0.9467) for specificity given the sensitivity.

### 3.5. Network Meta-Analysis of Radar Bands

**Figure 6:**
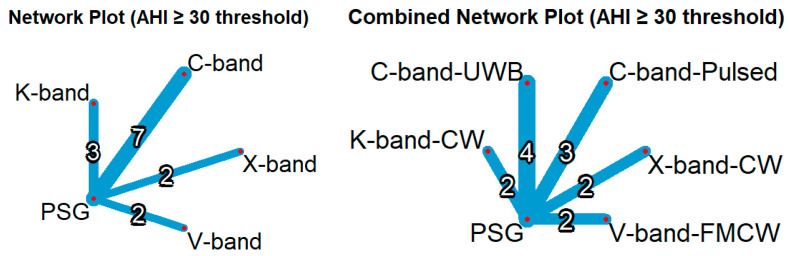
Network plot by frequency band and radar type. Left plot: Classified by frequency band. Right plot: Classified by the combination of frequency band and radar type. Nodes represent radar categories or PSG; connecting lines indicate direct comparisons, with numbers showing the number of studies for each comparison. Line width corresponds to the number of studies.

The network meta-analysis was conducted at AHI ≥30 events/h, based on the optimal AHI threshold of at least 22 events/h, and the analysis included 14 studies comparing five radar frequency bands (Figure 6). Among these, the X-band ranked the highest in diagnostic performance in both the common and random-effects models, followed by the K-band. In contrast, the C-band and V-band ranked lowest. Radar systems operating in the C-band, K-band, and V-band demonstrated consistently lower odds ratios (ORs; ranging from 0.0081 to 0.03; all *p* < 0.001). Conversely, the X-band yielded a substantially higher OR (0.1159; 95% CI: 0.0082–1.6365), although this finding did not reach statistical significance (*p* = 0.1106).

The combined network meta-analysis, which evaluated both radar frequency bands and radar types at AHI thresholds of 30 events/h or higher, included 13 studies, 13 pairwise comparisons, and 5 combinations of radar types and frequencies. In both the common and random effects models, C-band UWB and V-band FMCW showed the lowest ORs (ORs < 0.01, *p* < 0.0001), while X-band CW was the highest among radar types (common model OR: 0.1296, *p* = 0.0228; random model OR: 0.1171, *p* = 0.0956).

In both overall band-level and band-radar type analyses (Table 3), the X-band consistently ranked highest among radar modalities in both common and random effects. K-band followed with moderate *p* scores, whereas the C-band and V-band showed lower performances, with values declining further when paired with the radar type available in this meta-analysis. Heterogeneity remained moderate in both models (I^2^ = 67.3–70.4%), and inconsistency across designs was not detected.

### 3.6. Sensitivity Analysis

Following the adjustments of sensitivity analysis, pooled estimates in the sensitivity model showed only minor variations, consistent with expected threshold tradeoffs, and the overall diagnostic performance remained stable. Network rankings largely stayed consistent. Details of the sensitivity test are provided in Tables S24 and S25, Figures S9 and S10.

## 4. Discussion

Our meta-analysis synthesized diagnostic accuracy data from 19 studies on radar-based devices for OSA detection, focusing on per-patient or per-record AHI data across different frequency bands. Pooled sensitivity remained high across all thresholds, with at least moderate accuracy even at higher AHI levels of 30 events/h. At the optimal AHI threshold of ≥22 events/h, the X-band exhibited the highest consistency and accuracy, followed by the variable K-band, with the C-band exhibiting the lowest.

The superior performance of the X-band, especially when paired with CW radar, is likely attributable to its optimal balance between resolution and penetration depth for respiratory motion detection [59,60]. The X-band, however, exhibited a comparatively higher OR (0.1159, 95% CI: 0.0082–1.6365), which did not reach statistical significance (*p* = 0.1106), indicating greater diagnostic variability or reduced precision across the included studies. Alternatively, the C-band, despite its broad representation in these studies, was consistently associated with a low diagnostic OR (0.0081; 95% CI: 0.0022–0.0301) in the random-effects model and low P scores. The devices using C-band pulsed or UWB radar [57,61] might not offer sufficient resolution or robustness against interference in practical clinical settings. Despite high heterogeneity of this frequency-only model, the robustness of the findings is supported by significant z-scores (z = −7.18 for C-band; z = −3.59 for K-band; *p* < 0.001), and stability across the frequency-based performance hierarchy. In biomedical radar, frequency selection is a tradeoff: high frequencies (60–79 GHz) provide high resolution but suffer from limited range and interference, while low frequencies (sub-10 GHz) offer better penetration and robustness but lower diagnostic precision [27,70]. Choice should align with clinical goals and environmental constraints.

In clinical practice, X-band radar offers promise as a non-contact modality for sleep-disordered breathing assessment; however, its usability is strongly influenced by motion management strategies. Two studies using dual continuous-wave (CW) X-band radar systems operating at 10.525 GHz illustrate contrasting approaches. The 2016 system employed explicit motion cancellation via signal processing filters that removed low-frequency, large-amplitude components associated with random body movement (RBM) to isolate the small respiration signal [59]. By contrast, the 2018 system used hardware redundancy (two radars in separate positions) to recover signals lost due to posture or movement, which meant that RBM-affected periods were skipped rather than corrected [60]. While active motion cancellation supports uninterrupted data capture, it adds algorithmic complexity, and hardware redundancy may simplify design but risks data gaps [71,72]. These trade-offs underscore the need for optimized motion mitigation to ensure X-band radar’s reliability and practicality in diverse clinical environments.

The American Academy of Sleep Medicine (AASM) considers the AHI, particularly laboratory-based PSG, as the definitive severity measure [49,73]. In contrast, the respiratory event index (REI), an alternative index, yielded lower sensitivity (42.1% and 72.8%) and specificity (80.7% and 90.9%) than AHI-based ground truth [74] and consistently underestimated OSA severity, with a mean difference of −1.4 events/h [75]. The pooled metrics demonstrate that radar-based systems consistently achieved high sensitivity across all thresholds. However, specificity was more variable, being markedly lower at AHI ≥5 events/h and improving with increasing thresholds at AHI ≥30 events/h. Heterogeneity (I^2^) slightly increased with AHI thresholds, remaining within low to moderate ranges. To better capture diagnostic performances across various thresholds, a multiple-cutoff model revealed an optimal AHI threshold of 21.91 events/h, aligning with guidelines that suggest an AHI of 18 events/h from portable monitors corresponds to a PSG AHI of >10 events/h [76]. The model’s AUC of 0.91 meets the benchmark for OSA detection in uncomplicated adults [17].

The diagnostic performance summarized in this meta-analysis reflects not only the radar frequency band but also crucial methodological factors, including algorithmic support and sensor placement distance. Across the included studies, a clear evolution in computational aids was evident, from proprietary and rule-based algorithms (13 studies) to advanced ML and DL techniques (7 studies). Studies utilizing DL models, such as convoluted neural network (CNN), long- and short-term memory (LSTM) [63], attention-based architectures [68], and neural decision trees [65], demonstrated a high correlation with PSG AHI (*r* ≥ 0.91), compared to older rule-based systems [57]. Integration of advanced DL algorithms, such as attention-based networks and transformer architectures, is anticipated to further refine event-level classification and capture complex temporal dependencies in respiratory signals [68]. Moreover, the shift toward multi-modal data fusion, combining radar signals with pulse oximetry, audio, or movement data, is likely to reduce false positives and enhance classification robustness, particularly in home environments with variable noise and motion artifacts [55]. The high-dimensional electrophysiological data generated during PSG or from longitudinal health records are well-suited for artificial intelligence (AI), which facilitates precise and automated scoring of sleep and respiratory events and minimizes inter-scorer variability [77,78,79]. Still, AI studies have mostly remained narrowly trained on homogeneous datasets, which limits their cross-population effectiveness and increases the risk of erroneous classification when applied to heterogeneous clinical settings [80]. Furthermore, cloud-based platforms that allow for iterative model updates and clinician-in-the-loop feedback offer a promising route to ensure continuous performance optimization and clinical alignment [63,64,78].

In parallel, sensor-to-subject distance appeared to influence the diagnostic accuracy, with most studies placing sensors 0.2–2.0 m from participants, typically targeting the torso from either bedside or ceiling-mounted positions. Dual-sensor radar systems placed under the mattress or targeting the chest have shown high precision in capturing respiratory signals due to optimal alignment and proximity [59,62]. The overhead position outperformed the lateral one in standalone use, and combining both further improved specificity and reduced misclassifications [62]. In opposition, ceiling-mounted radars positioned beyond 1.5 m face increased signal attenuation and interference, potentially reducing accuracy unless mitigated by advanced algorithms, such as convolutional recurrent neural networks (CRNNs) [64]. At greater distances, the signal-to-noise ratio (SNR) drops, making it difficult to distinguish physiological signals, like respiration and heartbeat, from background interference [71,81]. The ability to filter out unwanted background signals (clutter suppression) becomes less effective past 2 m, and high-frequency systems, while more sensitive, are also more vulnerable to noise [82,83]. Therefore, maintaining a subject–sensor distance of 0.2–1.5 m in a combined direction may be suitable for the reliable detection of physiological signals in non-contact radar systems. However, variations in signal quality can occur due to factors such as radar design, environmental conditions, and signal processing methods [27,84].

In addition to the distance from the subject, bandwidth, and operating frequency, radar requires technical aspects, such as penetration depth, displacement resolution, and angular resolution [27,84]. X-band lower-frequency systems could effectively penetrate bedding and human tissues, ensuring reliable respiration monitoring in through-mattress setups [59,60]. However, its resolution may not capture subtle cardiac-induced chest wall motion. Conversely, higher-frequency systems, such as 24 GHz and 79 GHz (K- and W-band) radars, achieve higher spatial resolution, down to 0.1 mm in some cases, but with reduced penetration depth, making them more sensitive to subject distance and positional changes [54,65]. Intermediate frequencies, such as IR-UWB (>6 GHz), offer a balance, providing high resolution with adequate penetration for unobstructed or lightly obstructed configurations [43]. However, many studies did not report penetration depth or range resolution in standardized units [39,40,41,53,55,57,58,61,62,63,64,66,67,68,69], the degree to which these parameters affect pooled diagnostic estimates remains partly inferential. The absence of this information highlights the need for future studies to report such specifications explicitly to facilitate robust cross-technology comparisons.

This work offers a novel comparison of radar frequency bands for OSA detection. Strengths include comprehensive multi-database searching, strict inclusion of per-patient AHI data, and a consistent methodology using bivariate and HSROC models across validated AHI thresholds. Focusing the network meta-analysis at AHI ≥ 30 events/h, which aligns with the CICS-derived optimal threshold (≥22 events/h), provides a clinically meaningful benchmark. However, a major limitation is the lack of direct head-to-head comparisons between radar bands, relying solely on indirect evidence via PSG comparators, which may have introduced transitivity bias [85]. The QUADAS-2 assessment indicated unclear or selective patient recruitment in several studies, such as an enriched cohort with one-third of participants diagnosed with periodic limb movement in sleep (PLMS) [53], undefined eligibility criteria [54], unspecified recruitment methods [55], and absent selection details [68], may have introduced spectrum and selection biases. These factors could overestimate diagnostic accuracy, along with unclear index test interpretation and undisclosed or industry-linked funding. One study with only five cases was excluded from the meta-analysis. The small sample size counts per band, with only two studies for the X-band and heterogeneity in radar types and settings, further limit the generalizability. Although one study with only five cases was excluded to reduce small-sample bias [54], the remaining studies (18–196 participants) may still have limited statistical power. Publication bias, supported by Deeks’ funnel plot and statistical testing reduced certainty of evidence. Heterogeneity was low to moderate in pooled analyses (I^2^ = 10.7–17.8%) but higher in network meta-analyses at AHI ≥30 events/h (I^2^ = 67.3–70.4%), reflecting variability in radar technologies and study designs. These elements could compromise the strength and broader applicability of our conclusions.

The scope of radar frequency bands included in this meta-analysis was inherently limited by the availability of clinical validation studies, with most studies clustered around a narrow set of radar modalities and frequency bands (C-, X-, K-, V-, and W-bands). Notably, advanced radar configurations such as stepped-frequency CW (SFCW) and higher-frequency mmWave radars (110–300 GHz) remain underrepresented, despite their theoretical advantages in resolution and beamforming capacity [20,36,37,38,67]. Radar systems used in the included studies were operated within constrained frequency spectra, commonly ranging from 5.8 to 79 GHz, leaving higher bands (110–300 GHz) and hybrid modalities largely unexplored in clinical contexts.

Therefore, to ensure broader clinical adoption, future validation studies should aim to diversify radar configurations and expand the inclusion of underutilized frequency bands, especially in home-based and real-world sleep monitoring applications, to refine AHI estimations and ensure generalizability across populations. Furthermore, consensus on clinically meaningful, standardized AHI thresholds derived from radar (as opposed to traditional PSG AHI), will be essential for adjusting diagnostic decision-making and regulatory approval pathways. Ultimately, next-generation radar systems will likely evolve into wearable-free, AI-augmented platforms capable of longitudinal sleep monitoring and early identification of OSA phenotypes, with the potential for personalized risk stratification [65,86]. Future radar-based detection algorithms will presumably adopt end-to-end, event-level deep learning with multimodal fusion, particularly integrating radar respiratory features and SpO_2_, while incorporating boundary refinement, stage-aware priors, and motion-aware masking to enhance accuracy [55,62,63,64,65,67,68].

## 5. Conclusions

Radar-based systems demonstrated high sensitivity for OSA detection, with diagnostic accuracy influenced by the frequency band, AI support, and sensor proximity, which is optimal within a range of 0.2–1.5 m. X-band dual radars, paired with advanced algorithms, performed most consistently, followed by more-variable results from K-band radar; C-band radar demonstrated lower accuracy. Broader frequency exploration, standardized radar-derived AHI thresholds, and clinic-based validation are essential for future clinical integration.

## Figures and Tables

**Table 3:** P score ranking table for the network meta-analysis (AHI ≥ 30 events/h).

Treatment	P Score (Common)	P Score (Random)
PSG	0.9971	0.9861
X-band	0.736	0.6961
K-band	0.4964	0.5007
C-band	0.2577	0.2332
V-band	0.0128	0.0838
PSG	0.9977	0.9904
X-band-CW	0.7752	0.7308
C-band-Pulsed	0.5309	0.5265
K-band-CW	0.4926	0.5096
V-band-FMCW	0.0742	0.1426
C-band-UWB	0.1294	0.1002

PSG, polysomnography; CW, continuous wave; FMCW, frequency-modulated continuous wave; UWB, ultra-wide bandwidth.

## Data Availability

All data supporting the findings are included in the article and the Supplementary Documents.

## Supplementary Materials

Supplementary materials are available to download at https://www.mdpi.com/article/10.3390/diagnostics15162111/s1: Supplementary Documents, Figure S1: Summary receiver operating characteristic (SROC) curve; Figure S2: Deeks funnel plot (approximated) for AHI threshold set to 5 events/h; Figure S3: Deeks funnel plot (approximated) for AHI threshold set to 15 events/h; Figure S4: Deeks funnel plot (approximated) for AHI threshold set to 30 events/h; Figure S5: Forest plot and meta-analysis summary for X-band radar studies; Figure S6: Forest plot and meta-analysis summary for V-band radar studies; Figure S7: Forest plot and meta-analysis summary for K-band radar studies; Figure S8: Forest plot and meta-analysis summary for C-band radar studies; Figure S9: Network plot (AHI ≥ 30 events/h threshold) of sensitivity analyses; Figure S10: Combined network plot (AHI ≥ 30 events/h threshold) of sensitivity analyses; Table S1: PRISMA-DTA checklist of items; Table S2: PRISMA NMA checklist of items to include when reporting a systematic review involving a network meta-analysis; Table S3: PubMed search strategy; Table S4: Cochrane search strategy; Table S5: Diagnostic performance at different AHI cutoff thresholds; Table S6: Sensitivity and specificity with 95% CI (cut-off = 5); Table S7: Bivariate diagnostic random-effects meta-analysis (cut-off = 5); Table S8: Sensitivity and specificity with 95% confidence intervals (CIs) (cut-off = 15); Table S9: Bivariate diagnostic random-effects meta-analysis (cut-off = 15); Table S10: Sensitivity and specificity with 95% confidence intervals (CIs) (cut-off = 30); Table S11: Bivariate diagnostic random-effects meta-analysis (cut-off = 30); Table S12: Common effects model (band analysis); Table S13: Random effects model (band analysis); Table S14: Tests of heterogeneity (band analysis); Table S15: Data for AHI ≥ 30 events/h (band analysis); Table S16: Common effects pairwise results with Q and leverage metrics (band analysis); Table S17: Random Common effects pairwise results with Q and leverage metrics (band analysis); Table S18: Common effects model (band–wave interaction); Table S19: Random effects model (band–wave interaction); Table S20: Tests of heterogeneity (band–wave interaction); Table S21: Data comparisons with combinations (band–wave interaction); Table S22: Common effects pairwise results with Q and leverage metrics (band–wave interaction); Table S23: Random common effects pairwise results with Q and leverage metrics (band–wave interaction); Table S24: Analysis of frequency band (sensitivity analyses); Table S25: Analysis of combinations between frequency band and radar type (sensitivity analyses); Table S26: Distribution of included studies by radar frequency band and modulation technique; Table S27: Grading the evidence of the subgroup; Table S28: Critical appraisal of included studies using GRADE-informed domains. Reference [87] is cited in Supplementary Materials.

## Abbreviations

The following abbreviations are used in this manuscript:

AHI	Apnea–hypopnea index
AI	Artificial intelligence
AUC	Area under the curve
BW	Bandwidth
CI	Confidence interval
CW	Continuous wave
DL	Deep learning
fc	Center frequency
FMCW	Frequency-modulated continuous wave
FN	False negative
FP	False positive
HSAT	Home sleep apnea testing
IEEE	Institute of Electrical and Electronics Engineers
IRUWB	Impulse radio ultra-wideband
LFMCW	Linear frequency-modulated continuous wave
ML	Machine learning
OSA	Obstructive sleep apnea
PLMS	Periodic limb movement in sleep
PSG	Polysomnography
ROC	Receiver operating characteristic
SDI	Sleep disorder index
SFCW	Step frequency continuous wave
TN	True negative
TP	True positive
VHF	Very high frequency
