# Supplementary material for: Radar-Based Detection of Obstructive Sleep Apnea: A Systematic Review and Network Meta-Analysis of Diagnostic Accuracy Across Frequency Bands
Source: Diagnostics (Basel). 2025 Aug 21;15(16):2111. doi: 10.3390/diagnostics15162111 (PMC12385411; doi:10.3390/diagnostics15162111)
Supplement: Supplementary file 1 [file diagnostics-15-02111-s001.zip › diagnostics-3801367-Supplementary.pdf]

Supplementary Documents

Contents

1. PRISMA Diagnostic Test Accuracy (DTA) 2
2. PRISMA NMA Checklist of Items to Include When Reporting A Systematic Review Involving a Network Meta-analysis 5
3. Details of search strategy 9
   3.1. PubMed 9
   3.2. Cochrane 9
4. Selection criteria and data extraction 10
5. Multiple-cutoff models 10
6. Meta-analysis of radar-based detection of OSA at different AHI thresholds 11
   6.1. AHI threshold set to 5 events/h 11
   Bivariate diagnostic random-effects meta-analysis 12
   Deeks funnel plot (approximated) 12
   6.2. AHI Threshold set to 15 event/h 13
   Bivariate diagnostic random-effects meta-analysis 13
   Deeks funnel plot (approximated) 15
   6.3. AHI Threshold set to 30 events/h 15
   Bivariate diagnostic random-effects meta-analysis 16
   Deeks funnel plot (approximated) 16
7. Pairwise random-effects meta-analysis (AHI ≥ 30 events/h Threshold) 17
   7.1. Forest plot and meta-analysis summary for X-band radar studies 17
   7.2. Forest plot and meta-analysis summary for V-band radar studies 17
   7.3. Forest plot and meta-analysis summary for K-band radar studies 17
   7.4. Forest plot and meta-analysis summary for C-band radar studies 17
8. Network meta-analysis with an AHI threshold of 30 events/h 18
   8.1. Analysis of frequency bands 18
   8.2. Analysis of combination between frequency band and radar type 20
9. Sensitivity analyses 22
   9.1. Network meta-analysis with an AHI threshold of 30 events/h 22
   Analysis of frequency band 22
   Analysis of combinations between frequency band and radar type 22
10. Distribution of included studies by radar frequency band and modulation technique 23
11. Grading the certainty of the evidence (GRADE) 25

1. PRISMA Diagnostic Test Accuracy (DTA)

Table S1: PRISMA-DTA Checklist Item

Section/topic	#	PRISMA-DTA Checklist Item	Reported on page #
**TITLE / ABSTRACT**			
Title	1	Identify the report as a systematic review (+/- meta-analysis) of diagnostic test accuracy (DTA) studies.	1
Abstract	2	2.1. Identify the report as a systematic review (+/- meta-analysis) of diagnostic test accuracy (DTA) studies.<br>2.2. Indicate the research question, including components such as participants, index test, and target conditions.<br>2.3. Include study characteristics used as criteria for eligibility.<br>2.4. List the key databases searched and the search dates.<br>2.5. Indicate the methods of assessing risk of bias and applicability.<br>2.A1 Indicate the methods for the data synthesis.<br>2.6. Indicate the number and type of included studies and the participants and relevant characteristics of the studies (including the reference standard).<br>2.7. Include the results for the analysis of diagnostic accuracy, preferably indicating the number of studies and participants. Describe test accuracy including variability; if meta-analysis was done, include summary results and confidence intervals.<br>2.9. Provide a brief summary of the strengths and limitations of the evidence<br>2.10. Provide a general interpretation of the results and the important implications.<br>2.11. Indicate the primary source of funding for the review.<br>2.12. Provide the registration number and the registry name	1
**INTRODUCTION**			
Rationale	3	Describe the rationale for the review in the context of what is already known.	2
Clinical role of index test	D1	State the scientific and clinical background, including the intended use and clinical role of the index test, and if applicable, the rationale for minimally acceptable test accuracy (or minimum difference in accuracy for comparative design).	3
Objectives	4	Provide an explicit statement of question(s) being addressed in terms of participants, index test(s), and target condition(s).	2,3
**METHODS**			
Protocol and registration	5	Indicate if a review protocol exists, if and where it can be accessed (e.g., Web address), and, if available, provide registration information including registration number.	3
Eligibility criteria	6	Specify study characteristics (participants, setting, index test(s), reference standard(s), target condition(s), and study design) and report characteristics (e.g., years considered, language, publication status) used as criteria for eligibility, giving rationale.	3
Information sources	7	Describe all information sources (e.g., databases with dates of coverage, contact with study authors to identify additional studies) in the search and date last searched.	3
Search	8	Present full search strategies for all electronic databases and other sources searched, including any limits used, such that they could be repeated.	S9

Section/topic	#	PRISMA-DTA Checklist Item	Reported on page #
Study selection	9	State the process for selecting studies (i.e., screening, eligibility, included in systematic review, and, if applicable, included in the meta-analysis).	3, S10
Data collection process	10	Describe method of data extraction from reports (e.g., piloted forms, independently, in duplicate) and any processes for obtaining and confirming data from investigators.	3, S10
Definitions for data extraction	11	Provide definitions used in data extraction and classifications of target condition(s), index test(s), reference standard(s) and other characteristics (e.g. study design, clinical setting).	3, S10
Risk of bias and applicability	12	Describe methods used for assessing risk of bias in individual studies and concerns regarding the applicability to the review question.	3, S23-32
Diagnostic accuracy measures	13	State the principal diagnostic accuracy measure(s) reported (e.g. sensitivity, specificity) and state the unit of assessment (e.g. per-patient, per-lesion).	3
Synthesis of results	14	Describe methods of handling data, combining results of studies and describing variability between studies. This could include, but is not limited to: a) handling of multiple definitions of target condition. b) handling of multiple thresholds of test positivity, c) handling multiple index test readers, d) handling of indeterminate test results, e) grouping and comparing tests, f) handling of different reference standards	3
Meta-analysis	D2	Report the statistical methods used for meta-analyses, if performed.	3, S11-17
Additional analyses	16	Describe methods of additional analyses (e.g., sensitivity or subgroup analyses, meta-regression), if done, indicating which were pre-specified.	3,4
**RESULTS**			
Study selection	17	Provide numbers of studies screened, assessed for eligibility, included in the review (and included in meta-analysis, if applicable) with reasons for exclusions at each stage, ideally with a flow diagram.	3, 4
Study characteristics	18	For each included study provide citations and present key characteristics including: a) participant characteristics (presentation, prior testing), b) clinical setting, c) study design, d) target condition definition, e) index test, f) reference standard, g) sample size, h) funding sources	5
Risk of bias and applicability	19	Present evaluation of risk of bias and concerns regarding applicability for each study.	S23-32
Results of individual studies	20	For each analysis in each study (e.g. unique combination of index test, reference standard, and positivity threshold) report 2x2 data (TP, FP, FN, TN) with estimates of diagnostic accuracy and confidence intervals, ideally with a forest or receiver operator characteristic (ROC) plot.	S11-15
Synthesis of results	21	Describe test accuracy, including variability; if meta-analysis was done, include results and confidence intervals.	11,12,13, S11-17
Additional analysis	23	Give results of additional analyses, if done (e.g., sensitivity or subgroup analyses, meta-regression; analysis of index test: failure rates, proportion of inconclusive results, adverse events).	12, S20-21
**DISCUSSION**			
Summary of evidence	24	Summarize the main findings including the strength of evidence.	11,12

Section/topic	#	PRISMA-DTA Checklist Item	Reported on page #
Limitations	25	Discuss limitations from included studies (e.g. risk of bias and concerns regarding applicability) and from the review process (e.g. incomplete retrieval of identified research).	15,16
Conclusions	26	Provide a general interpretation of the results in the context of other evidence. Discuss implications for future research and clinical practice (e.g. the intended use and clinical role of the index test).	18
**FUNDING**			
Funding	27	For the systematic review, describe the sources of funding and other support and the role of the funders.	15, S23-32

Adapted From: McInnes, M.D.F.; Moher, D.; Thombs, B.D.; McGrath, T.A.; Bossuyt, P.M.; The PRISMA-DTA Group; Clifford, T.; Cohen, J.F.; Deeks, J.J.; Gatsonis, C.; et al. Preferred Reporting Items for a Systematic Review and Meta-analysis of Diagnostic Test Accuracy Studies: The PRISMA-DTA Statement. JAMA ***2018****, 319, 388-396. https://doi.org/10.1001/jama.2017.19163.*

2. PRISMA NMA Checklist of Items to Include When Reporting A Systematic Review Involving a Network Meta-analysis

Table S2: PRISMA NMA Checklist of Items to Include When Reporting A Systematic Review Involving a Network Meta-analysis

Section/Topic	Item #	Checklist Item	Reported on Page #
**TITLE**			
Title	1	Identify the report as a systematic review *incorporating a network meta-analysis (or related form of meta-analysis).*	1
**ABSTRACT**			
Structured summary	2	Provide a structured summary including, as applicable:<br>**Background:** main objectives<br>**Methods:** data sources; study eligibility criteria, participants, and interventions; study appraisal; and *synthesis methods, such as network meta-analysis.*<br>**Results:** number of studies and participants identified; summary estimates with corresponding confidence/credible intervals; *treatment rankings may also be discussed. Authors may choose to summarize pairwise comparisons against a chosen treatment included in their analyses for brevity.*<br>**Discussion/Conclusions:** limitations; conclusions and implications of findings.<br>**Other:** primary source of funding; systematic review registration number with registry name.	1
**INTRODUCTION**			
Rationale	3	Describe the rationale for the review in the context of what is already known*, including mention of why a network meta-analysis has been conducted.*	2
Objectives	4	Provide an explicit statement of questions being addressed, with reference to participants, interventions, comparisons, outcomes, and study design (PICOS).	2
**METHODS**			
Protocol and registration	5	Indicate whether a review protocol exists and if and where it can be accessed (e.g., Web address); and, if available, provide registration information, including registration number.	2
Eligibility criteria	6	Specify study characteristics (e.g., PICOS, length of follow-up) and report characteristics (e.g., years considered, language, publication status) used as criteria for eligibility, giving rationale. *Clearly describe eligible treatments included in the treatment network, and note*	2,3

		whether any have been clustered or merged into the same node (with justification).	
Information sources	7	Describe all information sources (e.g., databases with dates of coverage, contact with study authors to identify additional studies) in the search and date last searched.	3,4
Search	8	Present full electronic search strategy for at least one database, including any limits used, such that it could be repeated.	S9
Study selection	9	State the process for selecting studies (i.e., screening, eligibility, included in systematic review, and, if applicable, included in the meta-analysis).	3,4, S10
Data collection process	10	Describe method of data extraction from reports (e.g., piloted forms, independently, in duplicate) and any processes for obtaining and confirming data from investigators.	3,4, S10
Data items	11	List and define all variables for which data were sought (e.g., PICOS, funding sources) and any assumptions and simplifications made.	Table 1, S23-32
**Geometry of the network**	**S1**	Describe methods used to explore the geometry of the treatment network under study and potential biases related to it. This should include how the evidence base has been graphically summarized for presentation, and what characteristics were compiled and used to describe the evidence base to readers.	5-9,14, S23-32
Risk of bias within individual studies	12	Describe methods used for assessing risk of bias of individual studies (including specification of whether this was done at the study or outcome level), and how this information is to be used in any data synthesis.	10, S23-32
Summary measures	13	State the principal summary measures (e.g., risk ratio, difference in means). *Also describe the use of additional summary measures assessed, such as treatment rankings and surface under the cumulative ranking curve (SUCRA) values, as well as modified approaches used to present summary findings from meta-analyses.*	3,5,11,12
Planned methods of analysis	14	Describe the methods of handling data and combining results of studies for each network meta-analysis. This should include, but not be limited to:<br>• *Handling of multi-arm trials;*<br>• *Selection of variance structure;*<br>• *Selection of prior distributions in Bayesian analyses; and*<br>• *Assessment of model fit.*	3
**Assessment of Inconsistency**	**S2**	Describe the statistical methods used to evaluate the agreement of direct and indirect evidence in the treatment network(s) studied. Describe efforts taken to address its presence when found.	NA

Risk of bias across studies	15	Specify any assessment of risk of bias that may affect the cumulative evidence (e.g., publication bias, selective reporting within studies).	3,10
Additional analyses	16	Describe methods of additional analyses if done, indicating which were pre-specified. This may include, but not be limited to, the following:<br>• Sensitivity or subgroup analyses;<br>• Meta-regression analyses;<br>• *Alternative formulations of the treatment network; and*<br>• *Use of alternative prior distributions for Bayesian analyses (if applicable).*	S20-21
**RESULTS†**			
Study selection	17	Give numbers of studies screened, assessed for eligibility, and included in the review, with reasons for exclusions at each stage, ideally with a flow diagram.	Fig.1
**Presentation of network structure**	**S3**	Provide a network graph of the included studies to enable visualization of the geometry of the treatment network.	Fig. 6
**Summary of network geometry**	**S4**	Provide a brief overview of characteristics of the treatment network. This may include commentary on the abundance of trials and randomized patients for the different interventions and pairwise comparisons in the network, gaps of evidence in the treatment network, and potential biases reflected by the network structure.	S11-12
Study characteristics	18	For each study, present characteristics for which data were extracted (e.g., study size, PICOS, follow-up period) and provide the citations.	S22-32
Risk of bias within studies	19	Present data on risk of bias of each study and, if available, any outcome level assessment.	S23-32
Results of individual studies	20	For all outcomes considered (benefits or harms), present, for each study: 1) simple summary data for each intervention group, and 2) effect estimates and confidence intervals. *Modified approaches may be needed to deal with information from larger networks.*	S11-15
Synthesis of results	21	Present results of each meta-analysis done, including confidence/credible intervals. *In larger networks, authors may focus on comparisons versus a particular comparator (e.g. placebo or standard care), with full findings presented in an appendix. League tables and forest plots may be considered to summarize pairwise comparisons.* If additional summary measures were explored (such as treatment rankings), these should also be presented.	11-12, S11-17
**Exploration for inconsistency**	**S5**	Describe results from investigations of inconsistency. This may include such information as measures of model fit to compare consistency and inconsistency models, *P*	Table 2, S11-16

		values from statistical tests, or summary of inconsistency estimates from different parts of the treatment network.	
Risk of bias across studies	22	Present results of any assessment of risk of bias across studies for the evidence base being studied.	S23-32
Results of additional analyses	23	Give results of additional analyses, if done (e.g., sensitivity or subgroup analyses, meta-regression analyses*, alternative network geometries studied, alternative choice of prior distributions for Bayesian analyses,* and so forth).	S20-21
**DISCUSSION**			
Summary of evidence	24	Summarize the main findings, including the strength of evidence for each main outcome; consider their relevance to key groups (e.g., healthcare providers, users, and policy-makers).	11-12
Limitations	25	Discuss limitations at study and outcome level (e.g., risk of bias), and at review level (e.g., incomplete retrieval of identified research, reporting bias). *Comment on the validity of the assumptions, such as transitivity and consistency. Comment on any concerns regarding network geometry (e.g., avoidance of certain comparisons).*	16-17
Conclusions	26	Provide a general interpretation of the results in the context of other evidence, and implications for future research.	17
**FUNDING**			
Funding	27	Describe sources of funding for the systematic review and other support (e.g., supply of data); role of funders for the systematic review. This should also include information regarding whether funding has been received from manufacturers of treatments in the network and/or whether some of the authors are content experts with professional conflicts of interest that could affect use of treatments in the network.	18

PICOS = population, intervention, comparators, outcomes, study design.

* Text in italics indicateS wording specific to reporting of network meta-analyses that has been added to guidance from the PRISMA statement.

† Authors may wish to plan for use of appendices to present all relevant information in full detail for items in this section.

3. Details of search strategy

3.1. PubMed

Table S3: PubMed Search Strategy

PubMed	Query string
#1 non-contact sensors using radar	radar[All Fields] OR "interferometry"[tiab] OR "Doppler"[tiab] OR "frequency-modulated continuous wave"[tiab] OR FMCW[tiab] OR "ultrawideband"[tiab] OR UWB[tiab] OR IR-UWB[tiab] OR "radiofrequency"[tiab] OR "radio wave"[tiab] OR "Low Frequency"[tiab] OR "LF"[tiab] OR "L-band"[tiab] OR "S-band"[tiab] OR "C-band"[tiab] OR "X-band"[tiab] OR "Ku-band"[tiab] OR "K-band"[tiab] OR "Ka-band"[tiab] OR "mmWave"[tiab] OR "V-band"[tiab] OR "W-band"[tiab] OR "Ultra-Wideband"[tiab] OR "GHz"[tiab] OR "MHz"[tiab]
#2 Sleep	"Sleep"[MeSH] OR "Sleep Wake Disorders"[MeSH] OR "Respiratory Event Index"[Title/Abstract] OR "REI"[Title/Abstract] OR "apnea-hypopnea index"[Title/Abstract] OR "apnoea-hypopnea index"[Title/Abstract] OR "AHI"[Title/Abstract] OR "apnea-hypopnea"[Title/Abstract] OR "apnoea-hypopnea"[Title/Abstract] OR "apneahypopnea"[Title/Abstract] OR "respiratory disturbance index"[Title/Abstract] OR "RDI"[Title/Abstract] OR "oxygen desaturation index"[Title/Abstract] OR "ODI"[Title/Abstract] OR "mean oxygen saturation"[Title/Abstract] OR "lowest oxygen saturation"[Title/Abstract] OR "hypoxemia"[Title/Abstract] OR "oxygen desaturation"[Title/Abstract] OR "nocturnal hypoxia"[Title/Abstract] OR "sleep*"[Title/Abstract] OR "SDB"[Title/Abstract] OR "OSA"[Title/Abstract] OR "CSA"[Title/Abstract] OR "REM latency"[Title/Abstract] OR "arousal index"[Title/Abstract] OR "microarousal"[Title/Abstract] OR "WASO"[Title/Abstract] OR "polysomnograph*"[Title/Abstract] OR "actigraphy"[Title/Abstract] OR "snor*"[Title/Abstract] OR "gasping"[Title/Abstract] OR "morning headache"[Title/Abstract]
#3 Filters applied	Humans, Adult: 19+ years, Young Adult: 19-24 years, Adult: 19-44 years, Middle Aged + Aged: 45+ years, Middle Aged: 45-64 years, Aged: 65+ years, 80 and over: 80+ years.
#4	#1 AND #2 AND #3

3.2. Cochrane

Table S4: Cochrane Search Strategy

Step	Query string
#1	MeSH descriptor: [Sleep] explode all trees
#2	MeSH descriptor: [Sleep Wake Disorders] explode all trees
#3	("Respiratory Event Index" OR "REI" OR "Apnea-Hypopnea Index" OR "Apnoea-Hypopnea Index" OR "AHI" OR "Apnea-Hypopnea" OR "Apnoea-Hypopnea" OR "Apneahypopnea" OR "Respiratory Disturbance Index" OR "RDI" OR "Oxygen Desaturation Index" OR "ODI" OR "Mean Oxygen Saturation" OR "Lowest Oxygen Saturation" OR "Hypoxemia" OR "Oxygen Desaturation" OR "Nocturnal Hypoxia" OR "Sleep" OR "SDB" OR "OSA" OR "CSA" OR "ESS" OR "SSS" OR "Fatigue Severity Scale" OR "FSS" OR "EDS" OR "Fatigue" OR "Chronic Fatigue" OR "PSQI" OR "TST" OR "REM Latency" OR "Arousal Index" OR "Microarousal" OR "WASO" OR "Polysomnograph" OR "Actigraphy" OR "Snoring" OR "Episodes" OR "Gasping" OR "Morning Headache"):ti,ab,kw
#4	#1 OR #2 OR #3
#19	#17 OR #18
#20	#4 AND #16 AND #19

4. Selection criteria and data extraction

Studies were included if they assessed adult populations with suspected or confirmed obstructive sleep apnea (OSA) and utilized radar-based technologies generating patient-level apnea-hypopnea index (AHI) values. Only studies that used polysomnography (PSG) as the reference standard were eligible. Furthermore, inclusion was restricted to clinical validation studies that presented data at the individual patient level, allowing for the extraction of complete confusion matrix values—true positives, false positives, false negatives, and true negatives.

Studies were excluded if they lacked per-patient radar-derived AHI data, reported only event- or epoch-level outcomes, or did not address diagnostic relevance to OSA. Also excluded were studies focused solely on signal processing or physiological measurements without diagnostic classification, in vitro or simulation-only studies, and those without human participants. Abstract-only publications with incomplete data, duplicates, and prototype reports without clinical deployment were excluded. Additionally, studies limited to narrowly defined populations (e.g., heart failure only) were omitted to maintain generalizability to broader clinical settings.

For data extraction, when a single study reported multiple results derived from different analytical models, the result with the best diagnostic performance was selected for inclusion. Additionally, when studies evaluated multiple AHI thresholds, priority was given to standard or clinically accepted thresholds (such as AHI of ≥ 5, ≥ 10, ≥ 15, ≥ 20, ≥ 25, and ≥ 30 events/h) rather than data-driven or optimized thresholds, which might not be generalizable beyond the specific context of the study. Data on radar systems operating across various frequency bands (e.g., K-band, Ka-band, UWB) defined by IEEE and employing different techniques (e.g., Doppler, FMCW, IR-UWB) were also extracted.

5. Multiple-cutoff models

Table S5: Diagnostic Performance at Different AHI Cutoff Thresholds

Cutoff	Sensitivity	SE (Sens)	95% CI Sensitivity	Specificity	SE (Spec)	95% CI Specificity
5	0.9314	0.3914	[0.8630, 0.9669]	0.5962	0.4146	[0.3958, 0.7689]
10	0.8891	0.3529	[0.8006, 0.9412]	0.7595	0.3728	[0.6033, 0.8677]
15	0.8549	0.3492	[0.7482, 0.9211]	0.8313	0.3691	[0.7051, 0.9104]
20	0.8256	0.3555	[0.7023, 0.9048]	0.8711	0.3763	[0.7637, 0.9339]
25	0.7999	0.3653	[0.6614, 0.8911]	0.8962	0.3873	[0.8016, 0.9486]
30	0.7768	0.3762	[0.6247, 0.8792]	0.9134	0.3995	[0.8281, 0.9585]

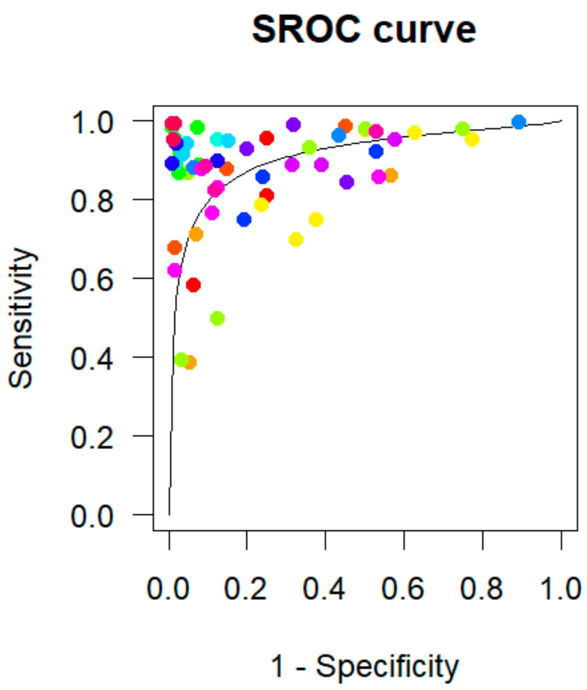

Figure S1: Summary Receiver Operating Characteristic (SROC) Curve

6. Meta-analysis of radar-based detection of OSA at different AHI thresholds

6.1. AHI threshold set to 5 events/h

Table S6: Sensitivity and Specificity with 95% CI (Cut-point = 5)

Study	TP	FP	FN	TN	Sensitivity (95% CI)	Specificity (95% CI)
Zhou 2020 [61]	113	0	0	63	1.00 [0.96–1.00]	1.00 [0.93–1.00]
Kwon 2021 [63]	30	0	0	6	1.00 [0.86–1.00]	1.00 [0.54–0.99]
Wei 2021 [40]	57	3	0	7	1.00 [0.92–1.00]	1.00 [0.40–0.89]
Choi 2022 [64]	35	4	0	5	1.00 [0.88–1.00]	0.56 [0.27–0.81]
Lin 2024 [65]	174	20	0	2	1.00 [0.97–1.00]	0.09 [0.03–0.28]
Anishchenko 2021 [62]	11	0	0	1	1.00 [0.69–1.00]	1.00 [0.13–0.96]
Li-Siheng 2024 [68]	9	1	0	8	1.00 [0.64–0.99]	0.89 [0.57–0.98]
Zaffaroni 2012 [58]	56	9	1	8	0.98 [0.91–1.00]	0.47 [0.26–0.69]
Weinreich 2017 [53]	30	15	1	11	0.97 [0.84–0.99]	0.42 [0.26–0.61]
Li 2021 [39]	50	2	2	17	0.96 [0.87–0.99]	0.89 [0.69–0.97]
Röcken 2025 [69]	79	9	6	8	0.93 [0.85–0.97]	0.47 [0.26–0.69]
Kang 2020 [43]	71	0	6	17	0.92 [0.84–0.96]	1.00 [0.78–1.00]
Wang 2024 [55]	66	3	7	24	0.92 [0.81–0.95]	0.89 [0.72–0.96]
Li-Chenyang 2024 [67]	94	2	12	37	0.89 [0.81–0.93]	0.95 [0.83–0.99]
Crinion 2019 [41]	46	8	7	6	0.87 [0.75–0.93]	0.43 [0.21–0.67]
Zaffaroni 2009 [57]	87	15	14	13	0.86 [0.78–0.92]	0.46 [0.30–0.64]

Bivariate diagnostic random-effects meta-analysis

Estimation method: restricted maximum likelihood (REML)

Table S7: Bivariate diagnostic random-effects meta-analysis (Cut-point = 5)

Term	Estimate	Std. Error	z value	Pr(>|z|)	95% CI Lower	95% CI Upper	Significance
tsens.(Intercept)	2.818	0.246	11.465	0	2.336	3.3	$p \leq 0.001$
tfpr.(Intercept)	-0.841	0.391	-2.152	0.031	-1.607	-0.075	$0.01 < p \leq 0.05$
sensitivity	0.944				0.912	0.964	
false pos. rate	0.301				0.167	0.481	

Variance Components (Between-Studies)

	Std. Dev.	Correlation with tsens	Correlation with tfpr
tsens	0.66	1	
tfpr	1.374	0.203	1

Partial AUC (restricted to observed FPRs and normalized): 0.937
I^2 estimates
Holling sample size unadjusted approaches: 33.7%–61.7%
Holling sample size adjusted approaches: 1.2%–3.4%

Deeks funnel plot (approximated)

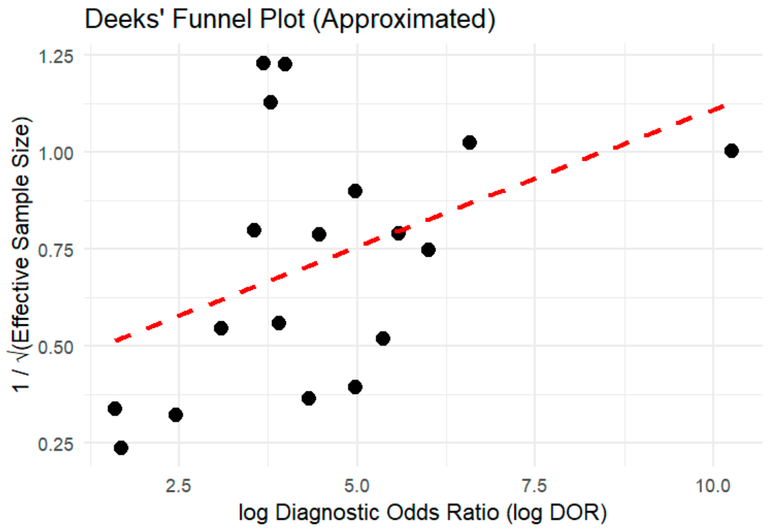

Figure S2: Deeks funnel plot (approximated) for AHI threshold set to 5 events/h

Residuals:

Min	1Q	Median	3Q	Max
-2.1505	-1.394	-0.1504	1.2023	5.0286

Coefficients:

Term	Estimate	Std. Error	t value	Pr(>|t|)	Significance
(Intercept)	2.525	1.085	2.327	0.0334	$0.01 < p \leq 0.05$

inv_sqrt_ESS	2.695	1.385	1.946	0.0695	.

Residual standard error: 1.844 with 16 degrees of freedom (df).
Multiple R^2: 0.1914; Adjusted R^2: 0.1408.
F-statistic: 3.786 on 1 and 16 df; p value: 0.06946.

6.2. AHI Threshold set to 15 event/h

Table S8: Sensitivity and Specificity with 95% Confidence Intervals (CIs) (Cut-point = 15)

Study	TP	FP	FN	TN	Sensitivity (95% CI)	Specificity (95% CI)
Zhou 2020 [61]	87	1	0	88	1.00 [0.95–1.00]	0.99 [0.94–1.00]
Li-Siheng 2024 [68]	5	0	0	13	1.00 [0.49–0.99]	1.00 [0.72–1.00]
Lin 2024 [65]	125	29	4	38	0.97 [0.92–0.99]	0.57 [0.45–0.68]
Kwon 2021 [63]	20	0	1	15	0.95 [0.77–0.99]	1.00 [0.75–1.00]
Weinreich 2017 [53]	28	8	3	18	0.90 [0.75–0.97]	0.69 [0.50–0.83]
Wang 2024 [55]	37	0	4	59	0.90 [0.77–0.96]	1.00 [0.92–1.00]
Li-Chenyang 2024 [67]	53	1	6	85	0.90 [0.80–0.95]	0.99 [0.94–1.00]
Kang 2020 [43]	44	3	5	42	0.90 [0.78–0.96]	0.93 [0.82–0.98]
Zaffaroni 2012 [58]	34	3	4	33	0.89 [0.76–0.96]	0.92 [0.78–0.97]
Choi 2022 [64]	25	2	3	14	0.89 [0.73–0.96]	0.88 [0.64–0.97]
Zaffaroni 2009 [57]	47	6	6	70	0.88 [0.77–0.95]	0.92 [0.84–0.96]
Gross-Isselmann 2024 [66]	36	1	5	58	0.88 [0.74–0.95]	0.98 [0.91–1.00]
Röcken 2025 [69]	51	10	8	33	0.86 [0.75–0.93]	0.77 [0.62–0.87]
Anishchenko 2021 [62]	6	1	1	4	0.86 [0.49–0.97]	0.80 [0.38–0.96]
Wei 2021 [40]	40	9	7	11	0.85 [0.72–0.94]	0.55 [0.34–0.74]
Gotoh_2016 [59]	5	4	1	14	0.83 [0.44–0.97]	0.78 [0.55–0.91]
Crinion 2019 [41]	23	2	9	33	0.72 [0.55–0.84]	0.94 [0.81–0.98]
Gotoh 2018 [60]	12	0	12	3	0.50 [0.31–0.69]	1.00 [0.36–0.98]

Bivariate diagnostic random-effects meta-analysis

Estimation method: restricted maximum likelihood (REML)

Table S9: Bivariate diagnostic random-effects meta-analysis (Cut-point = 15)

Term	Estimate	Std. Error	z value	Pr(>|z|)	95% CI Lower	95% CI Upper	Significance
tsens.(Intercept)	1.979	0.212	9.321	0	1.563	2.395	p ≤ 0.001
tfpr.(Intercept)	-2.164	0.337	-6.421	0	-2.825	-1.504	p ≤ 0.001
sensitivity	0.879				0.827	0.916	
false pos. rate	0.103				0.056	0.182	

Variance Components (Between-Studies)

	Std. Dev.	Correlation with tsens	Correlation with tfpr
tsens	0.665	1	
tfpr	1.176	0.041	1

Partial AUC (restricted to observed FPRs and normalized): 0.896
I^2 estimates
Holling sample size unadjusted approaches: 38.3%–54.5%
Holling sample size adjusted approaches: 1.8%–3.7%

Deeks funnel plot (approximated)

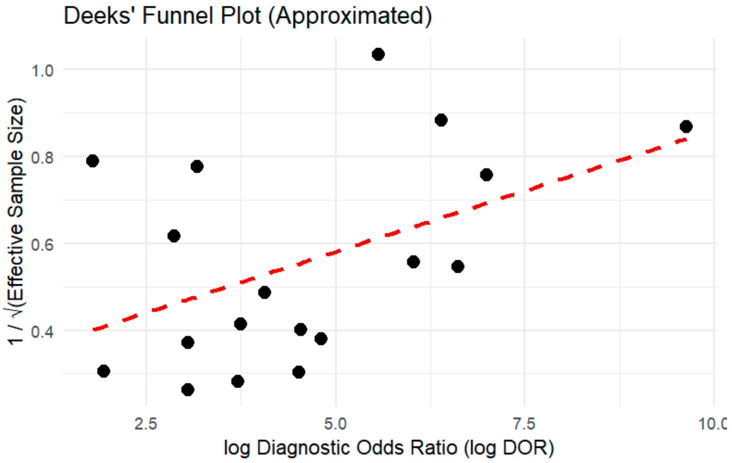

Figure S3: Deeks funnel plot (approximated) for AHI threshold set to 15 events/h

Residuals:

Min	1Q	Median	3Q	Max
-3.696	-0.858	-0.0202	0.92	3.843

Coefficients:

Term	Estimate	Std. Error	t value	Pr(>|t|)	Significance
(Intercept)	2.417	1.112	2.175	0.045	0.01 < p ≤ 0.05
inv_sqrt_ESS	3.884	1.839	2.113	0.0507	0.05 < p ≤ 0.1

Residual standard error: 1.824 with 16 degrees of freedom (d.f.).
Multiple R^2: 0.2181; Adjusted R^2: 0.1692.
F-statistic: 4.463 on 1 and 16 d.f.; p value: 0.05071.

6.3. AHI Threshold set to 30 events/h

Table S10: Sensitivity and Specificity with 95% Confidence Intervals (CIs) (Cut-point = 30)

Study	TP	FP	FN	TN	Sensitivity (95% CI)	Specificity (95% CI)
Kang 2020 [43]	33	0	0	61	1.00 [0.87–1.00]	1.00 [0.93–1.00]
Li-Chenyang 2024 [67]	33	1	1	110	0.97 [0.85–0.99]	0.99 [0.95–1.00]
Zhou 2020 [61]	53	1	2	120	0.96 [0.88–0.99]	0.99 [0.95–1.00]
Wang 2024 [55]	24	1	1	74	0.96 [0.90–0.99]	0.99 [0.93–1.00]
Wei 2021 [40]	33	6	2	26	0.94 [0.81–0.98]	0.81 [0.65–0.91]
Kwon 2021 [63]	11	0	1	24	0.92 [0.65–0.99]	1.00 [0.83–1.00]
Lin 2024 [65]	71	7	9	109	0.90 [0.78–0.97]	0.94 [0.80–0.99]
Zaffaroni 2012 [58]	16	6	3	49	0.84 [0.62–0.94]	0.89 [0.78–0.95]
Röcken 2025 [69]	25	13	8	56	0.76 [0.59–0.87]	0.81 [0.70–0.89]
Gotoh_2016 [59]	3	5	1	11	0.75 [0.30–0.95]	0.69 [0.44–0.86]
Choi 2022 [64]	9	0	4	31	0.72 [0.45–0.77]	1.00 [0.86–1.00]

Zaffaroni 2009 [57]	20	1	12	96	0.62 [0.45–0.77]	1.00 [0.94–1.00]
Anishchenko 2021 [62]	3	0	2	7	0.60 [0.23–0.88]	1.00 [0.58–0.99]
Gotoh 2018 [60]	5	0	8	14	0.50 [0.18–0.64]	1.00 [0.74–1.00]
Crinion 2019 [41]	8	2	13	44	0.38 [0.21–0.59]	0.96 [0.85–0.99]

Bivariate diagnostic random-effects meta-analysis

Estimation method: restricted maximum likelihood (REML)

Table S11: Bivariate diagnostic random-effects meta-analysis (Cut-point = 30)

Term	Estimate	Std. Error	z value	Pr(>|z|)	95% CI Lower	95% CI Upper	Significance
tsens.(Intercept)	1.566	0.37	4.237	0	0.841	2.29	p ≤ 0.001
tfpr.(Intercept)	-2.947	0.385	-7.654	0	-3.702	-2.192	p ≤ 0.001
sensitivity	0.827				0.699	0.908	
false pos. rate	0.05				0.024	0.1	

Variance Components: Between-Studies Std. Dev. and Correlation Matrix

	Std. Dev.	tsens	tfpr
tsens	1.219	1	—
tfpr	1.191	-0.298	1

Partial AUC (restricted to observed FPRs and normalized): 0.901

I^2 estimates
Holling sample size-unadjusted approaches: 60.2%–76%
Holling sample size-adjusted approaches: 5.3%–10.6%

Deeks funnel plot (approximated)

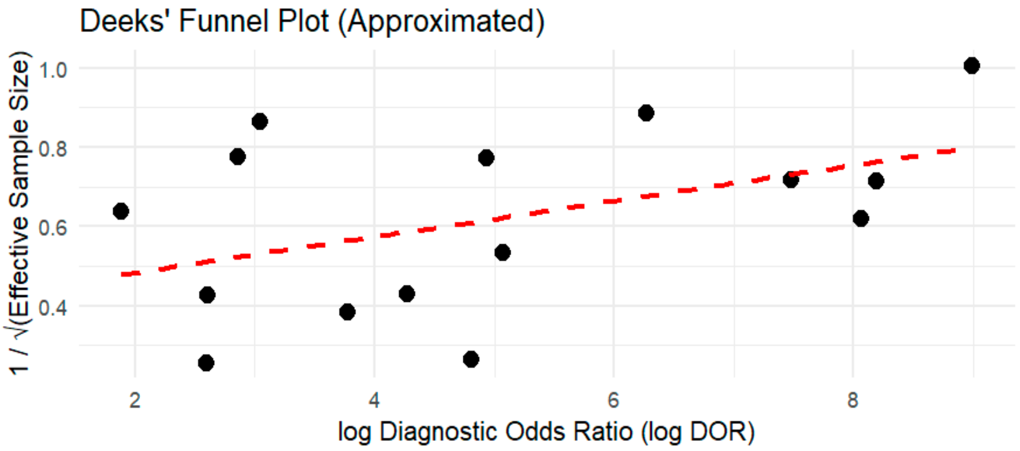

Figure S4: Deeks funnel plot (approximated) for AHI threshold set to 30 events/h

Residuals:

Min	1Q	Median	3Q	Max
-3.19	-1.134	0.0646	1.7	3.075

Coefficients:

Term	Estimate	Std. Error	t value	Pr(>|t|)	Significance
(Intercept)	2.178	1.633	1.333	0.2053	
inv_sqrt_ESS	4.55	2.485	1.831	0.0901	$0.05 < p \leq 0.1$

Residual standard error: 2.141 with 13 degrees of freedom (d.f.)
Multiple R^2: 0.205; Adjusted R^2: 0.1439
F-statistic: 3.353 on 1 and 13 d.f.; p value: 0.0901

7. Pairwise random-effects meta-analysis (AHI ≥ 30 events/h Threshold)

7.1. Forest plot and meta-analysis summary for X-band radar studies

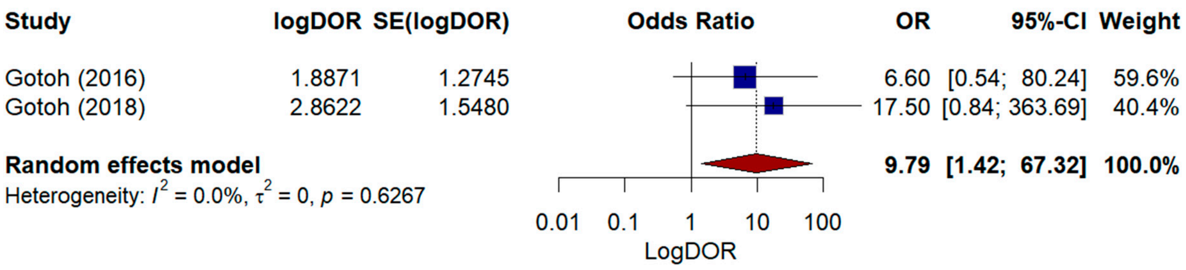

Figure S5: Forest plot and meta-analysis summary for X-band radar studies [59,60]

7.2. Forest plot and meta-analysis summary for V-band radar studies

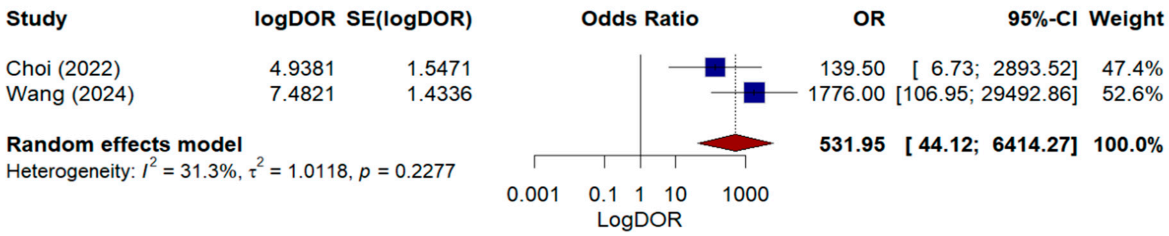

Figure S6: Forest plot and meta-analysis summary for V-band radar studies [55,64]

7.3. Forest plot and meta-analysis summary for K-band radar studies

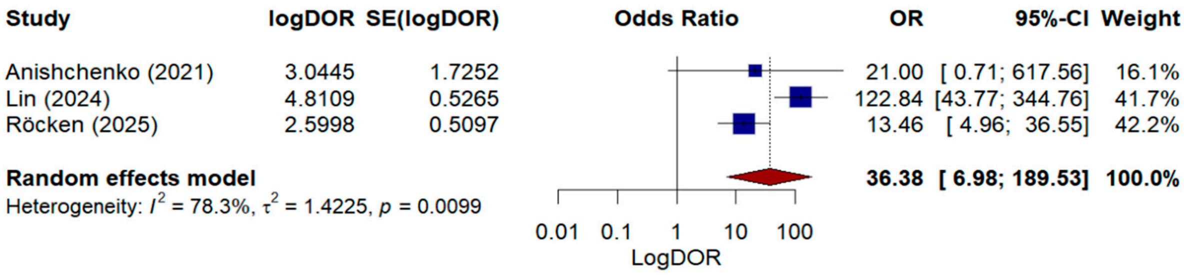

Figure S7: Forest plot and meta-analysis summary for K-band radar studies [62,65,69]

7.4. Forest plot and meta-analysis summary for C-band radar studies

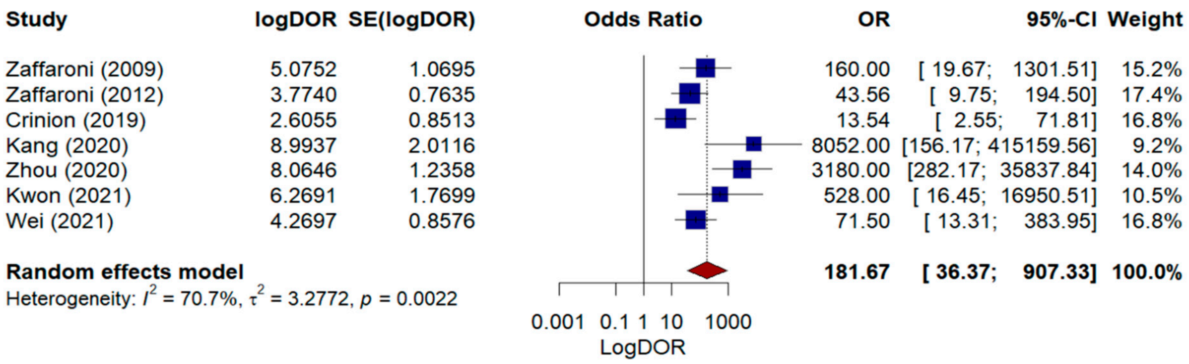

Figure S8: Forest plot and meta-analysis summary for C-band radar studies [40,41,43,57,58,61,63]

8. Network meta-analysis with an AHI threshold of 30 events/h

8.1. Analysis of frequency bands

Table S12: Common Effects Model (Band Analysis)

Treatment	OR	95% CI	z	p-value
C-band	0.0133	[0.0066; 0.0267]	-12.15	< 0.0001
K-band	0.0283	[0.0143; 0.0562]	-10.2	< 0.0001
PSG	–	–	–	–
V-band	0.0024	[0.0004; 0.0150]	-6.45	< 0.0001
X-band	0.1296	[0.0223; 0.7529]	-2.28	0.0228

Table S13: Random Effects Model (Band Analysis)

Treatment	OR	95% CI	z	p-value
C-band	0.0081	[0.0022; 0.0300]	-7.23	< 0.0001
K-band	0.03	[0.0047; 0.1914]	-3.71	0.0002
PSG	–	–	–	–
V-band	0.0027	[0.0002; 0.0398]	-4.31	< 0.0001
X-band	0.1159	[0.0082; 1.6365]	-1.6	0.1106

Quantifying heterogeneity / inconsistency:

$tau^2 = 1.9429$; $tau = 1.3939$; $I^2 = 70\%$ [44.1%; 83.8%]

Table S14: Tests of heterogeneity (Band Analysis)

Source	Q	d.f.	*p* value
Total	33.29	10	0.0002
Within designs	33.29	10	0.0002
Between designs	0	0	--

Table S15: Data for AHI ≥ 30 events/h (Band Analysis)

Study	treat1	treat2	TE	seTE
Zaffaroni 2009 [57]_AHI ≥ 30	C-band	PSG	-4.6588	0.8977
Zaffaroni 2012 [58]_AHI ≥ 30	C-band	PSG	-3.5808	0.7214
Crinion 2019 [41]_AHI ≥ 30	C-band	PSG	-2.4166	0.7832
Kang 2020 [43]_AHI ≥ 30	C-band	PSG	-9.0169	1.0115

Zhou 2020 [61]_AHI ≥ 30	C-band	PSG	-7.4496	1.0458
Kwon 2021 [63]_AHI ≥ 30	C-band	PSG	-5.9287	1.6717
Wei 2021 [40]_AHI ≥ 30	C-band	PSG	-4.0006	0.7883
Choi 2022 [64]_AHI ≥ 30	PSG	V-band	4.8903	1.536
Lin 2024 [65]_AHI ≥ 30	K-band	PSG	-4.6994	0.5116
Wang 2024 [55]_AHI ≥ 30	PSG	V-band	6.6985	1.178
Röcken 2025 [69]_AHI ≥ 30	K-band	PSG	-2.5302	0.4986
Gotoh_2016 [59]_AHI ≥ 30	PSG	X-band	1.5849	1.1051
Gotoh 2018 [60]_AHI ≥ 30	PSG	X-band	2.932	1.539
Anishchenko 2021 [62]_AHI ≥ 30	K-band	PSG	-3.0445	1.679

Table S16: Common-Effects Pairwise Results with Q and Leverage Metrics (Band Analysis)

Study	treat1	treat2	OR	95% CI	Q	Leverage
Zaffaroni 2009 [57]_AHI ≥ 30	C-band	PSG	0.0133	[0.0066; 0.0267]	0.14	0.16
Zaffaroni 2012 [58]_AHI ≥ 30	C-band	PSG	0.0133	[0.0066; 0.0267]	1.04	0.24
Crinion 2019 [41]_AHI ≥ 30	C-band	PSG	0.0133	[0.0066; 0.0267]	5.89	0.21
Kang 2020 [43]_AHI ≥ 30	C-band	PSG	0.0133	[0.0066; 0.0267]	5.46	0.03
Zhou 2020 [61]_AHI ≥ 30	C-band	PSG	0.0133	[0.0066; 0.0267]	8.97	0.12
Kwon 2021 [63]_AHI ≥ 30	C-band	PSG	0.0133	[0.0066; 0.0267]	0.93	0.05
Wei 2021 [40]_AHI ≥ 30	C-band	PSG	0.0133	[0.0066; 0.0267]	0.16	0.2
Choi 2022 [64]_AHI ≥ 30	PSG	V-band	415.26	[66.4788; 2593.9242]	0.3	0.37
Lin 2024 [65]_AHI ≥ 30	K-band	PSG	0.0283	[0.0143; 0.0562]	3.6	0.47
Wang 2024 [55]_AHI ≥ 30	PSG	V-band	415.26	[66.4788; 2593.9242]	0.32	0.63
Röcken 2025 [69]_AHI ≥ 30	K-band	PSG	0.0283	[0.0143; 0.0562]	4.29	0.49
Gotoh_2016 [59]_AHI ≥ 30	PSG	X-band	7.715	[1.3282; 44.8119]	0.17	0.19
Gotoh 2018 [60]_AHI ≥ 30	PSG	X-band	7.715	[1.3282; 44.8119]	0.33	0.34
Anishchenko 2021 [62]_AHI ≥ 30	K-band	PSG	0.0283	[0.0143; 0.0562]	0.1	0.04

Table S17: Random Common-Effects Pairwise Results with Q and Leverage Metrics (Band Analysis)

Study	treat1	treat2	OR	95% CI
Zaffaroni 2009 [57]_AHI ≥ 30	C-band	PSG	0.0081	[0.0022; 0.0300]
Zaffaroni 2012 [58]_AHI ≥ 30	C-band	PSG	0.0081	[0.0022; 0.0300]
Crinion 2019 [41]_AHI ≥ 30	C-band	PSG	0.0081	[0.0022; 0.0300]
Kang 2020 [43]_AHI ≥ 30	C-band	PSG	0.0081	[0.0022; 0.0300]
Zhou 2020 [61]_AHI ≥ 30	C-band	PSG	0.0081	[0.0022; 0.0300]
Kwon 2021 [63]_AHI ≥ 30	C-band	PSG	0.0081	[0.0022; 0.0300]
Wei 2021 [40]_AHI ≥ 30	C-band	PSG	0.0081	[0.0022; 0.0300]
Choi 2022 [64]_AHI ≥ 30	PSG	V-band	368.5372	[25.1328; 5404.0902]
Lin 2024 [65]_AHI ≥ 30	K-band	PSG	0.03	[0.0047; 0.1914]
Wang 2024 [55]_AHI ≥ 30	PSG	V-band	368.5372	[25.1328; 5404.0902]
Röcken 2025 [69]_AHI ≥ 30	K-band	PSG	0.03	[0.0047; 0.1914]
Gotoh_2016 [59]_AHI ≥ 30	PSG	X-band	8.6285	[0.6111; 121.8378]
Gotoh 2018 [60]_AHI ≥ 30	PSG	X-band	8.6285	[0.6111; 121.8378]

Anishchenko 2021 [62]_AHI ≥ 30	K-band	PSG	0.03	[0.0047; 0.1914]

8.2. Analysis of combination between frequency band and radar type

Number of studies: k = 13

Number of pairwise comparisons: m = 13

Number of treatments: *n* = 6

Number of designs: d = 5

Table S18: Common effects model (Band–Wave Interaction)

Treatment	OR	95% CI	z	*p* value
C-band-Pulsed	0.0313	[0.0128; 0.0766]	-7.58	< 0.0001
C-band-UWB	0.0036	[0.0012; 0.0109]	-9.95	< 0.0001
K-band-CW	0.0277	[0.0137; 0.0557]	-10.05	< 0.0001
PSG	–	–	–	–
V-band-FMCW	0.0024	[0.0004; 0.0150]	-6.45	< 0.0001
X-band-CW	0.1296	[0.0223; 0.7529]	-2.28	0.0228

Table S19: Random effects model (Band–Wave Interaction)

Treatment	OR	95% CI	z	*p* value
C-band-Pulsed	0.0295	[0.0054; 0.1614]	-4.06	< 0.0001
C-band-UWB	0.0022	[0.0004; 0.0128]	-6.77	< 0.0001
K-band-CW	0.027	[0.0041; 0.1798]	-3.74	0.0002
PSG	–	–	–	–
V-band-FMCW	0.0027	[0.0002; 0.0348]	-4.53	< 0.0001
X-band-CW	0.1171	[0.0094; 1.4583]	-1.67	0.0956

Quantifying heterogeneity / inconsistency:

$tau^2 = 1.6142$; tau = 1.2705; $I^2 = 67.1\%$ [33.7%; 83.7%]

Table S20: Tests of heterogeneity (Band–Wave Interaction)

Test Type	Q	d.f.	p-value
Total	24.35	8	0.002
Within designs	24.35	8	0.002
Between designs	0	0	--

Table S21: Data Comparisons with Combinations (Band–Wave Interaction)

Study	treat1	treat2	TE	seTE
Zaffaroni 2009 [57]_AHI ≥ 30	C-band-Pulsed	PSG	-4.6588	0.8977
Zaffaroni 2012 [58]_AHI ≥ 30	C-band-Pulsed	PSG	-3.5808	0.7214
Crinion 2019 [41]_AHI ≥ 30	C-band-Pulsed	PSG	-2.4166	0.7837

Kang 2020 [43]_AHI ≥ 30	C-band-UWB	PSG	-9.0169	2.0115
Zhou 2020 [61]_AHI ≥ 30	C-band-UWB	PSG	-7.4496	1.0458
Kwon 2021 [63]_AHI ≥ 30	C-band-UWB	PSG	-5.9287	1.6717
Wei 2021 [40]_AHI ≥ 30	C-band-UWB	PSG	-4.0006	0.7838
Choi 2022 [64]_AHI ≥ 30	PSG	V-band-FMCW	-4.8903	1.536
Lin 2024 [65]_AHI ≥ 30	K-band-CW	PSG	-4.6994	0.5116
Wang 2024 [55]_AHI ≥ 30	PSG	V-band-FMCW	6.6985	1.178
Röcken 2025 [69]_AHI ≥ 30	K-band-CW	PSG	-2.5302	0.4986
Gotoh_2016 [59]_AHI ≥ 30	PSG	X-band-CW	1.5849	1.1051
Gotoh 2018 [60]_AHI ≥ 30	PSG	X-band-CW	2.932	1.539

Table S22: Common-Effects Pairwise Results with Q and Leverage Metrics (Band–Wave Interaction)

**Study**	**treat1**	**treat2**	**OR**	**95%-CI**	**Q**	**leverage**
Zaffaroni 2009 [57]_AHI ≥ 30	C-band-Pulsed	PSG	0.0313	[0.0128; 0.0766]	1.77	0.26
Zaffaroni 2012 [58]_AHI ≥ 30	C-band-Pulsed	PSG	0.0313	[0.0128; 0.0766]	0.03	0.4
Crinion 2019 [41]_AHI ≥ 30	C-band-Pulsed	PSG	0.0313	[0.0128; 0.0766]	1.79	0.34
Kang 2020 [43]_AHI ≥ 30	C-band-UWB	PSG	0.0036	[0.0012; 0.0109]	2.84	0.08
Zhou 2020 [61]_AHI ≥ 30	C-band-UWB	PSG	0.0036	[0.0012; 0.0109]	3.04	0.29
Kwon 2021 [63]_AHI ≥ 30	C-band-UWB	PSG	0.0036	[0.0012; 0.0109]	0.03	0.11
Wei 2021 [40]_AHI ≥ 30	C-band-UWB	PSG	0.0036	[0.0012; 0.0109]	4.22	0.51
Choi 2022 [64]_AHI ≥ 30	PSG	V-band-FMCW	415.2601	[66.4788; 2593.9242]	0.55	0.37
Lin 2024 [65]_AHI ≥ 30	K-band-CW	PSG	0.0277	[0.0137; 0.0557]	4.73	0.49
Wang 2024 [55]_AHI ≥ 30	PSG	V-band-FMCW	415.2601	[66.4788; 2593.9242]	0.32	0.63
Röcken 2025 [69]_AHI ≥ 30	K-band-CW	PSG	0.0277	[0.0137; 0.0557]	4.69	0.51
Gotoh_2016 [59]_AHI ≥ 30	PSG	X-band-CW	7.715	[1.3282; 44.8119]	0.17	0.66
Gotoh 2018 [60]_AHI ≥ 30	PSG	X-band-CW	7.715	[1.3282; 44.8119]	0.33	0.34

Table S23: Random Common-Effects Pairwise Results with Q and Leverage Metrics (Band–Wave Interaction)

**Study**	**treat1**	**treat2**	**OR**	**95%-CI**
Zaffaroni 2009 [57]_AHI ≥ 30	C-band-Pulsed	PSG	0.0295	[0.0054; 0.1614]
Zaffaroni 2012 [58]_AHI ≥ 30	C-band-Pulsed	PSG	0.0295	[0.0054; 0.1614]

Crinion 2019 [41]_AHI ≥ 30	C-band-Pulsed	PSG	0.0295	[0.0054; 0.1614]
Kang 2020 [43]_AHI ≥ 30	C-band-UWB	PSG	0.0022	[0.0004; 0.0128]
Zhou 2020 [61]_AHI ≥ 30	C-band-UWB	PSG	0.0022	[0.0004; 0.0128]
Kwon 2021 [63]_AHI ≥ 30	C-band-UWB	PSG	0.0022	[0.0004; 0.0128]
Wei 2021 [40]_AHI ≥ 30	C-band-UWB	PSG	0.0022	[0.0004; 0.0128]
Choi 2022 [64]_AHI ≥ 30	PSG	V-band-FMCW	372.556	[28.7151; 4833.6218]
Lin 2024 [65]_AHI ≥ 30	K-band-CW	PSG	0.027	[0.0041; 0.1798]
Wang 2024 [55]_AHI ≥ 30	PSG	V-band-FMCW	372.556	[28.7151; 4833.6218]
Röcken 2025 [69]_AHI ≥ 30	K-band-CW	PSG	0.027	[0.0041; 0.1798]
Gotoh_2016 [59]_AHI ≥ 30	PSG	X-band-CW	8.5429	[0.6857; 106.4289]
Gotoh 2018 [60]_AHI ≥ 30	PSG	X-band-CW	8.5429	[0.6857; 106.4289]

9. Sensitivity analyses

9.1. Network meta-analysis with an AHI threshold of 30 events/h

Analysis of frequency band

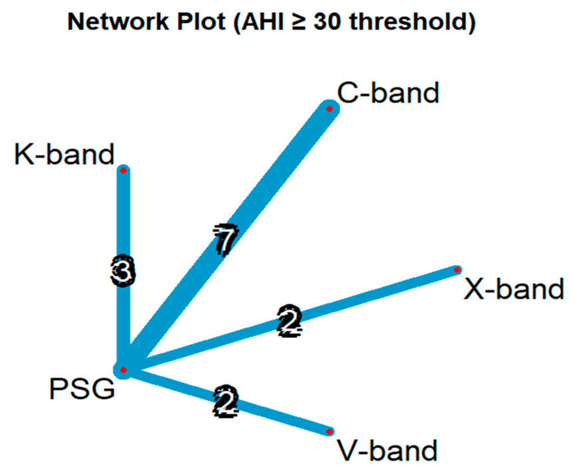

Figure S9: Network plot (AHI ≥ 30 events/h Threshold) of Sensitivity analyses

Table S24: Analysis of frequency band (Sensitivity analyses)

Treatment	P-score (common)	P-score (random)
PSG	0.9971	0.9859
X-band	0.7353	0.6846
K-band	0.4897	0.4907
C-band	0.2307	0.1873
V-band	0.0472	0.1516

Analysis of combinations between frequency band and radar type

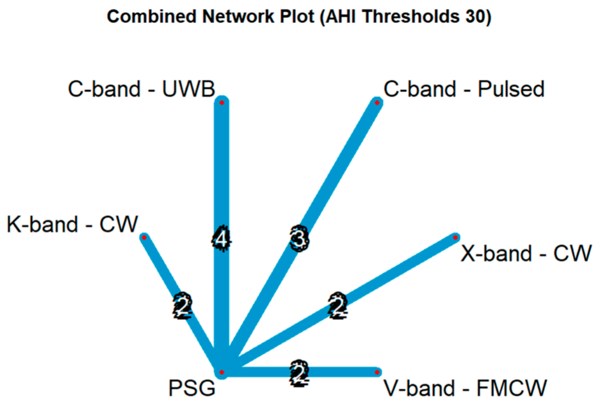

Figure S10: Combined network plot (AHI ≥ 30 events/h Threshold) of Sensitivity analyses

Table S25: Analysis of combinations between frequency band and radar type (Sensitivity analyses)

Method	P-score (common)	P-score (random)
PSG	0.9977	0.9904
X-band - CW	0.7753	0.7273
C-band - Pulsed	0.5313	0.515
K-band - CW	0.4811	0.4846
V-band - FMCW	0.1459	0.2181
C-band - UWB	0.0686	0.0645

10. Distribution of included studies by radar frequency band and modulation technique

Table S26: Distribution of included studies by radar frequency band and modulation technique

Band	Number of Studies	Pulsed	CW	FMCW	UWB	IR-UWB	Not Specified
**C band** (4–8 GHz)	9 studies	Zaffaroni 2009 [57], Zaffaroni 2012 [58], Crinion 2019 [41], Wei 2021 [40]			Weinreich 2017 [53]	Kang 2020 [43], Zhou 2020 [61], Kwon 2021 [63], Li-Siheng 2024 [68]	
**X band** (8–12 GHz)	2 studies		Gotoh 2016 [59], Gotoh 2018 [60]				
**K band** (18–27 GHz)	4 studies		Lin 2024 [65], Gross-Isselmann 2024 [66], Röcken 2025 [69]				Anishchenko 2021 [62]
**V band** (40–75 GHz)	2 studies			Choi 2022 [64], Wang 2024 [55]			
**W band** (75–110 GHz)	1 study			Koda 2023 [54]			

**mmWave** (110-300 GHz)	1 study	Li-Chenyang 2024 [67]

11. Grading the certainty of the evidence (GRADE)

Table S27: Grading the evidence of the subgroup

Threshold of AHI	Number of studies	Risk of Bias	Indirectness	Imprecision	Heterogeneity	Imprecision	Completeness	Certainty of Evidence (GRADE)	Reason(s) for downgrading
AHI ≥ 5	16	Not serious	Not serious	Not serious	Not serious	Not serious	Strongly detected	Moderate	Completeness
AHI ≥ 15	17	Not serious	Not serious	Not serious	Not serious	Not serious	Strongly detected	Moderate	Completeness
AHI ≥ 30	15	Not serious	Not serious	Not serious	Not serious	Not serious	Strongly detected	Moderate	Completeness

Customized QUADAS-2 visual plot: executed in a Jupyter Notebook v7.0.8)

Table S28: Critical appraisal of included studies using GRADE-informed domains

Lead Author, year	Risk of Bias	Applicability (Indirectness)	Heterogeneity (Inconsistency)	Imprecision	Completeness (Publication Bias)
**Zaffaroni 2009 [57]**	Patient selection: 159 consecutively admitted patients; 2 excluded (1 tech failure, 1 very short sleep).<br>Timing: SleepMinder and PSG recorded simultaneously.<br>Reference standard: Full PSG scored by expert.<br>SleepMinder: Used a proprietary, pre-specified signal processing pipeline.<br>Flow: Well reported, minimal attrition.	Population: Adults with suspected SDB; age 20–81 years, BMI 21–44 kg/m^2.<br>Setting: Sleep lab.<br>Index test: SleepMinder (5.8 GHz RF sensor).<br>Comparator: PSG (manual).<br>Thresholds: AHI ≥ 5, 10, 15, and 20 events/h.<br>Relevance: High — estimates AHI, a clinically relevant target.	AHI ≥ 5 events/h: Sens 86.1%, Spec 46.4%, AUC 0.858.<br>AHI ≥ 15 events/h: Sens 88.7%, Spec 92.1%, AUC 0.971.<br>AHI ≥ 30 events/h: Evaluated in severity class table.<br>Plots: ROC, Bland-Altman, scatter.<br>Stratified results: 4 severity levels; misclassification only within adjacent class.	Sample size: 129 subjects (test set).<br>Subgroup sizes: 35 normal, 55 mild, 28 moderate, 39 severe.<br>Performance metrics (Sens, Spec, AUC) provided for each threshold.<br>CIs not shown numerically; correlation reported as 91% CI (87.9%–93.8%).<br>2×2 table data: Not explicitly shown.	Funding: Not explicitly stated.<br>COI: Not declared, but 3 authors are from device company (BiancaMed).<br>Language: English.<br>Selective reporting: No major concerns.<br>Registration: Not mentioned.

**Zaffaroni 2012 [58]**	Patient selection: 75 patients recruited consecutively from a sleep clinic; 1 excluded due to short sleep (<45 min).<br>Exclusion: pregnant women, COPD requiring supplemental oxygen, those with previously diagnosed OSA.<br>Timing: Simultaneous PSG and SleepMinder recording.<br>Reference standard: Full PSG, scored by expert using AASM rules.<br>SleepMinder used a fully automated, pre-specified algorithm.<br>Flow: Well-described, minimal missing data.	Population: Adults (mean age 50 years), 80% male, 55% obese; suspected OSA.<br>Setting: Sleep lab.<br>Index test: SleepMinder (non-contact RF sensor, 5.8 GHz).<br>Comparator: PSG.<br>Thresholds used: AHI ≥ 5, ≥ 15, and ≥ 30 events/h.<br>Relevance: High — real-world clinical OSA detection.	AHI ≥ 5 events/h: Sens 98%, Spec 47%, AUC 0.90.<br>AHI ≥ 15 events/h: Sens 90%, Spec 92%, AUC 0.97.<br>AHI ≥ 30 events/h: Sens 84%, Spec 89%, AUC 0.96.<br>ROC curves, Bland-Altman plot, and severity classification matrix included.<br>Minimal misclassification; only 1 subject >1 severity class difference.	Sample size: 74 analyzed.<br>Severity distribution: 18.9% no OSA, 28.4% mild, 25.7% moderate, 27% severe.<br>2×2 data not provided, but full diagnostic metrics reported.<br>CIs for AUCs not shown but correlation $r = 0.90$ ($p < 0.0001$); strong agreement.	Funding: Devices provided free by BiancaMed (device company).<br>COI: 4 authors affiliated with BiancaMed;<br>COI declared.<br>Language: English.<br>Registration: Not reported.<br>Selective reporting: Low concern.
**Gotoh 2016 [59]**	- 20 patients with suspected SAS who underwent PSG as instructed by physicians.<br>- Clear inclusion/exclusion criteria.<br>- PSG and radar recorded simultaneously.<br>- PSG scored by certified technicians, Data analysis was outsourced to Teijin Pharma.<br>- Radar data were automatically calculated offline using software. Radar events were identified based on a 3-step algorithm.<br>- Flow clearly reported.	- Adult population with suspected SAS (age 35–67 years)<br>- Hospital sleep lab setting.<br>- Index test: microwave radar system (MVM).<br>- Comparator: PSG.<br>- AHI threshold: used for diagnosis; severity stratified.	- Sens and PPV for each subject at each threshold.<br>- Data for mild, moderate, severe SAS patients.<br>- ROC curve or CI not reported.<br>- No forest/prediction intervals.<br>- No subgroup analysis.	- Total sample size: 20.<br>- Full TP, FN data.<br>- CI not provided.<br>- Single-center study only.	- Funding disclosed (Kanagawa Innovation Project) (public funding).<br>- COI: Not reported, but authors include affiliated engineers.<br>- No trial registration.<br>- Results presented transparently.<br>- Published in Japanese with English abstract.
**Weinreich 2017 [53]**	- Authors designed a selected cohort including 1/3 PLMS patients.<br>- Patient selection: 57 patients recruited prospectively; 31 OSA, 19 PLMS, 6 CSR.	- Population: Adults, mean age 56.4 years, 46 males/11 females; comorbidities like HTN, CHF.<br>Setting: Sleep lab.<br>- Index test: SleepMinder (5.8 GHz non-contact sensor).	- Thresholds tested: AHI ≥ 5, 10, and 15 events/h; SDI ≥ 5, 10, and 15.<br>- Sens/Spec: Provided with 95% CIs for all thresholds.	- Sample size: 57 subjects.<br>2×2 tables: Not shown, but all diagnostic accuracy metrics (Sens, Spec, PPV, NPV, LR) provided.<br>- CIs: Reported for AHI and SDI thresholds.	- Funding: No external funding.<br>- COI: Declared — authors state no conflict – 1 author from ResMed, 1 received grant from ResMed.

	- Inclusion criteria: Suspected sleep disorder; enriched sample to include PLMS.<br>- Timing: Simultaneous SleepMinder and PSG.<br>- Reference standard: PSG with full AASM scoring.<br>- PSG data were analyzed by experienced investigators.<br>- The researchers interpreted the device-generated movement index.<br>- Flow: 57 analyzed; no attrition.	Comparator: PSG. Thresholds: AHI ≥ 5, 10, and 15 events/h; also SDI (AHI + PLMI) ≥ 5, 10, and 15.<br>Relevance: High; real-world mixed sleep disorder population.	- ROC and Bland-Altman plots provided for both AHI and SDI.<br>- Subgroup: SDI improved accuracy in PLMS subgroup.	- Per-threshold analysis: AHI and SDI thresholds analyzed separately.	- Language: English.<br>- Selective reporting: Low concern.<br>Registration: Not reported.
**Gotoh 2018 [60]**	- Researchers conducted clinical tests of the proposed system.<br>- 27 suspected SAHS patients (mean age 49 ± 12 years).<br>- Inclusion/exclusion: Not explicitly described.<br>- Simultaneous PSG and radar.<br>- PSG scored per AASM 2012, PSG data were scored by a third-party commercial service (Teijin Pharma).<br>- Radar data were scored automatically using an Excel-based algorithm, designed by the research team.<br>- Clear test flow.	- Adults suspected of SAHS cohort.<br>- Hospital sleep center setting.<br>- Dual Doppler radar system (MVM, TAU GIKEN Co. Ltd. Yokohama, Japan).<br>- PSG comparator.<br>- Thresholds AHI ≥15 and ≥30 events/h included.	- Sens/spec reported for AHI ≥15 and ≥30 events/h.<br>- ROC curves provided (pre/post optimization).<br>- No prediction intervals.<br>- No subgroup analysis.	- Sample size: 27.<br>- Radar event counts per patient provided.<br>- CIs: not reported.<br>- Single center.<br>- No breakdown of AHI categories per subject shown.	- Funding reported (Kanagawa government).<br>- COI: Declared — authors state no conflict.<br>- No registration.<br>- Full transparency in result reporting.<br>- Language: English.<br>- Selective reporting: Results appear complete for AHI evaluation.

**Crinion 2019 [41]**	- 67 from sleep clinic, 52 from HTN clinic.<br>- Inclusion/exclusion: Adults with suspected OSA/poor signal or technical issues. HTN cohort included only males.<br>- Timing: Simultaneous recording of index and reference tests.<br>- Reference standard: PSG (clinic) and polygraphy (home), scored per AASM.<br>- PSG was scored using AASM 2012 rules by trained sleep technologists.<br>- Radar data were automatically scored by a proprietary algorithm (developed by BiancaMed/ ResMed).<br>-Flow: Out of 125 enrolled: 119 included, 3 excluded (1 tech issue, 2 short recordings), 3 dropped out (sleep clinic cohort). Minor attrition (4.8%).	- Population: Adults aged 52–58 years, majority male (71%–100%), suspected OSA.<br>Setting: Both home (HTN cohort) and hospital (sleep clinic).<br>- Index test: SleepMinder (5.8 GHz biomotion sensor).<br>- Comparator: PSG (sleep clinic) or PG (HTN).<br>Thresholds: AHI ≥ 15 events/h used throughout.<br>- Relevance: High — evaluates ambulatory detection of moderate/severe OSA.	- Thresholds: Evaluated at AHI ≥ 5, 15, and 30 events/h.<br>- Performance metrics: Sens, Spec, AUC, PPV, NPV provided.<br>- ROC, Bland-Altman plots, kappa coefficients, & confusion matrices presented.<br>- Gender subgroup analysis included.	- Sample size: Total *n* = 122 (67 sleep clinic, 55 HTN).<br>- Events: Exact TP/FP/FN/TN not given, but Sens, Spec, PPV, NPV, κ reported for each subgroup.<br>- CIs shown for AUCs. Stratified by location (home/lab), population (clinic/HTN), and gender.<br>- Subgroup analysis: By cohort (sleep clinic vs. HTN).	- Funding: No external funding.<br>- COI: Not reported, but several authors affiliated with ResMed (SleepMinder manufacturer).<br>- Language: English.<br>- Selective reporting: Low concern - all major outcomes reported.<br>- Registry: Not mentioned.
**Kang 2020 [43]**	- Patient selection: 94 adults with suspected OSA who visited a sleep center were enrolled in the study.<br>- Exclusions: 5 due to short sleep time.<br>- Timing: Simultaneous IR-UWB radar and PSG recordings.<br>Reference standard: PSG, AASM scored.<br>- PSG: Manually scored by a trained sleep technician using AASM guidelines.<br>- Radar data: scored automatically using a custom algorithm developed by the research team.	- Population: Adults, range of severity (23 normal, 24 mild, 14 moderate, 33 severe OSA).<br>- Setting: Sleep lab.<br>Index test: IR-UWB radar (XK300-SA, Xandar Kardian).<br>- Comparator: PSG.<br>- Thresholds: AHI ≥ 5, ≥ 15, ≥ 30 events/h.<br>Relevance: High — real-world clinical population and clinically relevant thresholds.	- AHI ≥ 5 events/h: Sens 100%, Spec 92%, Agreement 0.93.<br>- AHI ≥ 15 events/h: Sens 93%, Spec 84%, Agreement 0.91.<br>- AHI ≥ 30 events/h: Sens 100%, Spec 100%, Agreement 1.0. ROC curves, Bland-Altman plots, and confusion matrices shown. Subgroup analysis by OSA severity provided.	- Sample size: 94 subjects. Events distributed across severity groups.<br>- Metrics: Sens, Spec, PPV, NPV, ICC = 0.927 (95% CI: 0.894–0.950).<br>- Limits of Agreement: Narrow in normal/mild; wider in severe OSA. Data stratified by severity.	- Funding: National Research Foundation (NRF), South Korea.<br>- COI: Declared — authors state no conflict, author affiliated with radar manufacturer.<br>- Language: English.<br>- Registration: Not stated.<br>- Selective reporting: Low concern.

	- Flow: 94 included, well documented.				
**Zhou 2020 [61]**	- Patient selection: Consecutive patients & volunteers from 3 sleep centers. Exclusion criteria: Severe comorbidities, BMI > 45 kg/m^2, inability to comply.<br>- Radar AHI calculated automatically by embedded chip.<br>- PSG data: manually scored & interpreted by certified physicians.<br>- Timing: Simultaneous PSG and radar throughout full night. Reference standard: Full PSG, AASM 2012 scoring manual. Flow: 176 included, no missing data.	- Population: Adults (mean age 38 years), 125 males, 51 females; mix of normal and OSA patients.<br>- Setting: Hospital sleep labs. Index test: UWB radar (ZG-S01A).<br>- Comparator: PSG (Respironics Alice5).<br>- Thresholds: AHI ≥ 5, 15, and 30 events/h.<br>- Relevance: High — directly aligned with OSA screening goals.	- Sens/Spec:<br>AHI ≥ 5 events/h: Sens 100%, Spec 100%, AUC 1.00.<br>AHI ≥ 15 events/h: Sens 97%, Spec 96%, AUC 0.99.<br>AHI ≥ 30 events/h: Sens 95%, Spec 99%, AUC 0.99.<br>- ROC, Bland-Altman, ICC, and subgroup severity concordance presented.	- Sample size: *n* = 176 (63 normal, 113 OSA).<br>- Subgroup counts: Normal (63), mild (26), moderate (32), severe (55).<br>- Metrics: Sens, Spec, PPV, NPV, ICC = 0.98 (CI 0.98–0.99), Kendall τ = 0.834, all CIs reported. Bland-Altman LOA: –13.04 to +10.99.	- Funding: Government sources (Zhejiang Province Health and Wuyi County Science Bureaus).<br>- COI: Declared — authors state no conflict.<br>- Language: English.<br>- Selective reporting: Low risk.<br>- Registration: Not mentioned.
**Anishchen-ko 2021 [62]**	- Inclusion/exclusion described (31 volunteers total, 27 with OSAS).<br>- PSG and radar recorded simultaneously overnight.<br>- PSG scored per AASM by somnologist.<br>- Radar data: scored automatically by machine learning classifiers, trained & validated by the research team using MATLAB.<br>- Flow reported (train/test = 19/12).	- Adults with suspected OSAS.<br>- Sleep lab setting (Almazov National Medical Center).<br>- Index test: dual bioradar (BioRASCAN-24).<br>- Comparator: PSG.<br>- AHI thresholds ≥5, 15, and 30 events/h used.	- Sens, Spec, accuracy, Cohen's kappa reported for AHI ≥ 15 events/h.<br>- Confusion matrices provided.<br>- No forest plot/prediction interval.<br>- No subgroup stratification.	- Total *N* = 31.<br>- Training: 19, Testing: 12.<br>- Confusion matrix allows TP, FP, FN, TN inference.<br>- No CI reported.	- Funding: declared, Supported by the Russian Science Foundation (RFBR).<br>- COI: Not reported.<br>- Language: English (IEEE proceedings).<br>- Registration: Not mentioned explicitly.

**Kwon 2021 [63]**	- Patient selection: adults ≥18 years from high-risk SAHS group (Berlin & STOP-Bang) were recruited.<br>- Inclusion/exclusion criteria clearly defined.<br>- PSG scored by technician, verified by 2 clinicians.<br>- Radar data were scored entirely by a hybrid CNN-LSTM deep learning architecture.<br>- Time interval: Simultaneous IR-UWB radar and PSG overnight.<br>- Flow: 40 recruited, 36 analyzed (4 excluded due to device issue).	- Population: suspected SAHS patients (age, sex, severity in Table 1).<br>- Setting: Sleep Center, Seoul National University Hospital.<br>- Index test: IR-UWB radar (Novelda X4) + CNN-LSTM.<br>- Comparator: PSG.<br>- Thresholds: AHI ≥5, ≥15, and ≥30 events/h used.<br>- Aligned with clinical SAHS diagnosis.	- Sens/Spec reported for AHI ≥5, 15, and 30 events/h.<br>- Kappa and stratified results by severity included.<br>- 3 thresholds (AHI ≥5, ≥15, and ≥30 events/h) evaluated.<br>- Variability discussed: performance increases with SAHS severity.	- Sample size: 36 subjects.<br>- Number of segments: 916,538 test segments.<br>- Events: TP, FP, FN, TN evaluated per segment.<br>- CI width not explicitly reported, but correlation (*r*=0.97) and Bland-Altman bias (−1.983) presented.<br>- Number of studies: 1 (single-center study).	- Funding: Supported by National Research Foundation (NRF), Korea MSIT.<br>- COI: Not reported.<br>- Language: English.<br>- Registry: mentioned explicitly.
**Li 2021 [39]**	- Inclusion/exclusion criteria well-defined. Cases (*n*=71) were randomly selected.<br>- Simultaneous PSG & radar monitoring.<br>- PSG scored per AASM by trained technicians.<br>- Radar data were scored automatically by the device's internal analysis software.<br>- Participant flow clearly reported.	- Adults with suspected OSA (age 18–76 years), both sexes.<br>- Hospital sleep center setting.<br>- Index test: new non-contact radar + SpO_2 ring.<br>- Comparator: PSG - AHI ≥ 5 events/h used for diagnosis; severity strata reported.	- AHI thresholds: ≥5 events/h and stratified by severity (mild, moderate, severe).<br>- Sens, Spec, ROC curve, and Bland-Altman analysis included.<br>- CI width: Not reported.<br>- No subgroup analysis by demographics.	- Sample size = 71.<br>- TP/FP/FN/TN clearly shown.<br>- CI not explicitly reported.<br>- Single-center study only.	- Funding: disclosed (Air Force Medical University).<br>- COI: Not reported.<br>- Language: Chinese with English abstract.<br>- No trial registration reported.<br>- All diagnostic data transparently presented.
**Wei 2021 [40]**	- Patient selection: Adults with suspected OSA from sleep center were assessed over the period of September 2018 to April 2019.<br>- Inclusion/exclusion criteria provided.<br>- PSG scored by technicians.<br>- Radar data: automatically scored by the UWB system software.<br>- Time interval: UWB and PSG conducted simultaneously.<br>- Flow: 67 enrolled, no dropouts reported.	- Age: Mean 43±11 years; both sexes (mostly males).<br>- Setting: Sleep center in China.<br>- Index test: UWB radar (Zhaoguan) + ring oximeter.<br>- Comparator: PSG.<br>- Thresholds: AHI ≥5, 15, and 30 events/h analyzed.	- Sens/Spec for AHI ≥5/15/30 events/h reported (Table 3).<br>- Kappa, ROC curve, Bland-Altman, and correlation (*r*=0.82) provided.<br>- No summary ROC plots.<br>- Subgroup by OSA severity included.<br>- Some over/underestimation noted in severe OSA due to position changes.	- *N* = 67 total.<br>- Subgroup sizes: 10 (non), 10 (mild), 12 (mod), 35 (severe).<br>- Sens/Spec per group reported.<br>- CI widths not provided explicitly.	- Funding: Reported (Liaoning Province Distinguished Professor grant).<br>- COI: Declared — authors state no conflict.<br>- Language: English.<br>- Selective reporting: Unclear, but key outcomes were reported.<br>- Registry or protocol: Not mentioned.

**Choi 2022 [64]**	- Clear inclusion/exclusion criteria (sleep-disordered breathing; excluded comorbid pulmonary/ cardiovascular/ cerebrovascular diseases).<br>- Patients were recruited from adults with chief complaints of sleep-disordered breathing.<br>- PSG and radar conducted simultaneously.<br>- PSG: Scored manually per AASM guidelines; exact scorers not specified.<br>- Radar: Labeled based on PSG, then scored using an automated deep learning model (CRNN).<br>- Participant flow: 55 recruited, 44 completed.<br>- Validated PSG (AASM 2012) used.	- Adults referred to sleep clinic with OSA symptoms.<br>- Setting: sleep lab.<br>- Index test: 60 GHz FMCW radar + CRNN.<br>- Comparator: full PSG.<br>- Thresholds: AHI ≥ 5, 15, and 30 events/h.<br>- Matches typical clinical diagnostic scenario.	- AUC, Sens, Spec reported across severity levels.<br>- ROC curves and Bland-Altman analyses provided.<br>- Subgroup performance: normal, mild, mod, severe OSA.<br>- Sens and false positives reported per subgroup.	- Sample size: 44 patients.<br>- Subgroups: 9 normal, 7 mild, 15 moderate, 13 severe OSA.<br>- Sens, Spec, kappa reported.<br>- CI for multiple performance metrics.	- Funding: Korean Government Medical Device Fund disclosed.<br>- COI: Declared — authors state no conflict, 3 authors employed by radar company (AU Inc.).<br>- Language: English.<br>- Registry/protocol: Approved by IRB, no public registry ID cited.
**Koda 2023 [54]**	- Simultaneous PSG and radar.<br>- Clear flow; 5 patients.<br>- Inclusion/Exclusion criteria: Not explicitly defined.<br>- Threshold was not pre-specified.<br>- PSG: scored by medical doctor/ sleep specialist.<br>- Radar scoring was done automatically by the system the researchers implemented.	- 5 adults with mild/moderate SAS.<br>- Hospital setting.<br>- Index: mm-wave FMCW radar (79 GHz) with expectation-maximization (EM) algorithm.<br>- Comparator: PSG.<br>- Threshold AHI ≥ 5 events/h.	- AHI errors reported per patient.<br>- Comparison with PSG in time segments.<br>- No ROC plots.	- *N*=5.<br>- TP/FP/FN/TN not directly stated.<br>- AHI error reported (mean error: 4.8 events/h).<br>- CI width: Not reported.<br>- Pilot-level sample.	- Funding sources listed (JST, JSPS, SECOM).<br>- No registration or protocol declaration.<br>- COI: Not reported.
**Lin 2024 [65]**	- Adult patients aged 20–80 years with suspected OSA: referred to the sleep center for PSG & were invited to participate.<br>- Clear inclusion & exclusion criteria reported.<br>- Index test (radar) & reference standard (PSG) performed simultaneously.	- Study population reflects typical OSA demographics.<br>- Setting was a hospital sleep lab, suitable for diagnostic evaluation.<br>- Index test: wireless 24 GHz CW radar with deep learning.<br>- Comparator: full PSG.	- Results presented for AHI thresholds of 5, 15, and 30 events/h.<br>- Sens, Spec, AUC, accuracy reported per threshold.<br>- Bland-Altman plots included.	- Total sample size of 196 with balanced OSA severity groups.<br>- Event numbers (TP, FP, FN, TN) provided via confusion matrix.<br>- CI reported for AUC.<br>- Single-center study with no replication.	- Funding source disclosed (Ministry of Science & Technology, Taiwan).<br>- Authors declared no COI.<br>- Language: English.<br>- Registry/protocol: Approved by IRB.

	- PSG scored independently by 2 technologists using AASM guidelines.<br>- Radar data: processed & scored by a machine learning system.<br>- Minimal missing data, total sample size 196.	- AHI thresholds evaluated: 5, 15, and 30 events/h.<br>- Highly relevant to the clinical question of OSA assessment.			
**Gross-Isselman 2024 [66]**	- Adult participants with suspected OSA or healthy volunteers: enrolled within an 11-month period.<br>- Clear inclusion/exclusion criteria and ethics-approved.<br>- PSG scored by 3 independent sleep technicians.<br>- Radar AHI: calculated automatically by the Sleepiz One + algorithm.<br>- Simultaneous data collection of PSG and radar device.<br>- 141 enrolled, 100 included in final analysis after exclusions.<br>- Reference standard: full PSG per AASM guidelines.	- Study population: 100 adults, both sexes, broad age range (mean age 52 years).<br>- Sleep center in Germany.<br>- Index test: 24 GHz radar-based Sleepiz One+.<br>- Comparator: PSG with AASM 2.6.<br>- AHI thresholds: primarily ≥ 15 events/h used for performance metrics.<br>- Relevant to clinical screening for moderate-to-severe OSA.	- Sens and Spec at AHI ≥ 15 events/h reported with and without SpO_2.<br>- ROC correlation (r = 0.87–0.94).<br>- Bland-Altman plots included.<br>- One AHI threshold analyzed (AHI ≥ 15 events/h) for classification.	- Final sample size: 100.<br>- TP, FP, FN, TN counts provided.<br>- Sens and Spec with CI implied but not fully reported.<br>- Only one threshold (AHI ≥ 15 events/h) used for evaluation.<br>- No replication in external settings.	- Study registered on ClinicalTrials.gov (NCT04670848).<br>- Funding disclosed (Sleepiz AG support, Project DEAL).<br>- COI: Declared — authors state no conflict.<br>- Language: English.<br>- Results comprehensively reported in main and supplemental files.
**Wang 2024 [55]**	- Recruitment method (random/consecutive) not described.<br>- Inclusion: adults undergoing PSG, Exclusion criteria not reported.<br>- Simultaneous data collection of PSG and radar device.<br>- PSG data scored by Sleep technologists.<br>- Radar data scored by deep learning models.<br>- 100 participants enrolled; no mention of dropouts.	- Adults with suspected OSAHS.<br>- Setting: sleep lab/hospital.<br>- Index test: 60 GHz FMCW radar & pulse oximeter (ROSA system).<br>- Reference test: PSG.<br>- AHI thresholds: 5, 15, and 30 events/h used for stratification.<br>- Highly relevant to clinical diagnostic setting.	- Sens, Spec, accuracy reported for AHI ≥ 5, 15, and 30 events/h.<br>- ROC curve described, ICC (0.9870) reported.<br>- Confusion matrix and severity-level metrics included.<br>- Subgroup results by severity group reported.	- Sample size: 100 subjects.<br>- Balanced across AHI severity groups (normal, mild, moderate, severe).<br>- TP, FP, FN, TN available.<br>- CI included for accuracy metrics.<br>- Analysis done per AHI threshold.	- Funding disclosed (National Natural Science Foundation of China).<br>- Trial registered (NCT06038006).<br>- COI: not explicitly stated.<br>- Comprehensive results reporting in main text and tables.
**Li-Chenyan 2024 [67]**	- Patient selection: from July to September 2023, 155 suspected OSA patients were recruited.	- Study population: Adults with suspected OSA, aged ≥18 years.	- Sens, Spec, accuracy, kappa reported for AHI ≥ 5, 15, and 30 events/h.	- Total N = 145.	- Trial registered (NCT06038006).

	- Clear inclusion/exclusion criteria.<br>- Simultaneous radar (QSA600) & PSG recording.<br>- PSG scored by 2 certified experts per AASM.<br>- Radar (QSA600) data were scored automatically using AI-based algorithms.<br>- Flow: 155 enrolled, 145 analyzed, reasons for exclusion listed.	- Conducted in hospital sleep lab - Index test: QSA600 (60GHz mmWave radar + oximeter).<br>- Comparator: PSG.<br>- Thresholds: AHI ≥ 5, 15, and 30 events/h.<br>- Directly aligned with clinical diagnosis of OSA.	- ROC curves with AUC.<br>- ICC, Pearson *r*, and Bland-Altman plots provided.<br>- Stratified by severity groups (mild, moderate, severe).	- Balanced severity groups (39 non-OSA, 47 mild, 25 moderate, 34 severe).<br>- TP, FP, FN, TN data implicit in Sens/Spec tables.<br>- CI for all diagnostic metrics reported.<br>- All 3 thresholds analyzed.	- Funding sources disclosed (STI2030, Shanghai grants, Tsinghua).<br>- COI: Declared — authors state no conflict.<br>- Language: Chinese (abstract in English).<br>- Results transparently presented.
**Li-Siheng 2024 [68]**	- Patient selection method: not reported; Mixed—hospital (*N* =18), ages 5–58 years; home (*N* = 7), aged 22–34 years.<br>- Inclusion/exclusion: Not clearly stated.<br>- Timing: Simultaneous PSG & radar.<br>- PSG data: scored by medical technicians.<br>- Radar data: scored by deep learning model.<br>- Flow: All participants included; no attrition reported.	- Adults with various AHI levels (normal to severe OSA).<br>- Setting: hospital-based sleep monitoring.<br>- Index: UWB radar with attention-reinforced model.<br>- Comparator: PSG. Thresholds: AHI ≥ 5, 15, and 30 events/h.<br>- Relevance: High — addresses clinical OSA screening in-lab.	- Performance at AHI thresholds: – AHI ≥ 5 events/h: accuracy 90.3% – AHI ≥ 15 events/h: 94.6% – AHI ≥ 30 events/h: 93.7%.<br>- ROC, Bland-Altman, confusion matrix reported.<br>- Subgroup (severity) results shown.<br>- Case/control breakdown: In-hospital: 9 healthy, 9 OSA with severity gradation.	- *N* = 24.<br>- Confusion matrix available; TP, FP, FN, TN calculable.<br>- No CIs reported for Sens/Spec. Stratification by AHI class shown.	- Funding: Declared - supported by Chinese national and provincial research funds.<br>- COI: Not reported.<br>- Language: English.<br>- Selective reporting: Low concern.<br>- Registration: Not mentioned.
**Röcken 2025 [69]**	- 120 recruited between May 2020 and January 2021.<br>- Clear inclusion/exclusion criteria.<br>- PSG and radar recorded simultaneously.<br>- PSG scored by certified physicians.<br>- Radar data: analyzed independently and externally by the company (Sleepiz AG, Switzerland).<br>- 102 participants analyzed after excluding ineligible cases.	- Adult patients with clinical suspicion of OSA.<br>- Hospital-based setting.<br>- Index test: SleepizOne+ (Doppler radar).<br>- Comparator: PSG.<br>- AHI thresholds: 5, 15, and 30 events/h used.<br>- Directly aligned with diagnostic purpose of OSA detection.	- ROC AUC reported for AHI ≥ 5, 15, and 30 events/h (0.92, 0.87, 0.89).<br>- Sens, Spec, PPV, NPV reported per threshold.<br>- Bland-Altman and Lin's concordance coefficient included.<br>- No forest plots or prediction intervals.<br>- Stratified by severity (mild/moderate/severe).	- *N* = 102 patients.<br>- Diagnostic thresholds all evaluated.<br>- Sens/Spec with 95% CI provided.<br>- TP/FP/FN/TN values derived from confusion matrices.<br>- Balanced severity groups included.	- Trial registered (ISRCTN45778591).<br>- Funding disclosed (Projekt DEAL).<br>- COI: Declared — authors state no conflict.<br>- Published in English.<br>- All methods and results clearly reported.

Spec, specificity; Sens, sensitivity; SDB, Sleep disordered breathing; BMI, body-mass index; RF, radio frequency; PSG, polysomnography; RERAs, respiratory effort-related arousals; COI, conflict of interest; CI, confidence interval; AHI, apnea-hypopnea index; AUC, area under the curve; ROC, receiver operating characteristic; HTN, hypertension; CHF, congestive heart failure; PLMS, periodic limb movements in sleep; CSR, Cheyne-Stokes respiration; AASM, American Academy of Sleep Medicine; COPD, chronic obstructive pulmonary disease; OSA, obstructive sleep apnea; SAS, sleep apnea syndrome; TP, true positive, TN, true negative; FP, false positive; FN, false negative; PLMS, periodic limb movements in sleep; PPV, positive predictive value; NPV, negative predictive value; LR, likelihood ratio; SDI, Sleep Disorder Index; PLMI, period limb movement index; SAHS, sleep apnea-hypopnea syndrome; PG, Polygraphy; IR-UWB, infrared ultra-wide bandwidth; ICC, intraclass correlation coefficients; OSAS, Obstructive sleep apnea syndrome; CNN-LSTM, convolutional neural network–long- and short-term memory; CRNN, convolutional recurrent neural network; FMCW, frequency-modulated continuous wave; OSAHS, Obstructive sleep apnea-hypopnea syndrome.
